# Challenges and Perspectives in Homology-Directed Gene Targeting in Monocot Plants

**DOI:** 10.1186/s12284-019-0355-1

**Published:** 2019-12-19

**Authors:** Tien Van Vu, Yeon Woo Sung, Jihae Kim, Duong Thi Hai Doan, Mil Thi Tran, Jae-Yean Kim

**Affiliations:** 1 Division of Applied Life Science (BK21 Plus program), Plant Molecular Biology and Biotechnology Research Center, Gyeongsang National University, Jinju, 660-701 Republic of Korea; 2 National Key Laboratory for Plant Cell Biotechnology, Agricultural Genetics Institute, Km 02, Pham Van Dong Road, Co Nhue 1, Bac Tu Liem, Hanoi, 11917 Vietnam; 3 Division of Life Science, Gyeongsang National University, 501 Jinju-daero, Jinju, 52828 Republic of Korea

**Keywords:** Gene targeting (GT), Homology-directed repair (HDR), Homology-directed gene targeting (HGT), CRISPR/Cas, Targeted mutagenesis, Precision breeding, Monocots

## Abstract

Continuing crop domestication/redomestication and modification is a key determinant of the adaptation and fulfillment of the food requirements of an exploding global population under increasingly challenging conditions such as climate change and the reduction in arable lands. Monocotyledonous crops are not only responsible for approximately 70% of total global crop production, indicating their important roles in human life, but also the first crops to be challenged with the abovementioned hurdles; hence, monocot crops should be the first to be engineered and/or de novo domesticated/redomesticated. A long time has passed since the first green revolution; the world is again facing the challenge of feeding a predicted 9.7 billion people in 2050, since the decline in world hunger was reversed in 2015. One of the major lessons learned from the first green revolution is the importance of novel and advanced trait-carrying crop varieties that are ideally adapted to new agricultural practices. New plant breeding techniques (NPBTs), such as genome editing, could help us succeed in this mission to create novel and advanced crops. Considering the importance of NPBTs in crop genetic improvement, we attempt to summarize and discuss the latest progress with major approaches, such as site-directed mutagenesis using molecular scissors, base editors and especially homology-directed gene targeting (HGT), a very challenging but potentially highly precise genome modification approach in plants. We therefore suggest potential approaches for the improvement of practical HGT, focusing on monocots, and discuss a potential approach for the regulation of genome-edited products.

## Background

### Status of Food Production Using Monocots

Most of the present important crop plants were domesticated approximately 10,000–13,000 years ago by our ancestors. The domestication of food crops forever changed human life from hunting-gathering groups to stationary living communities (Meyer and Purugganan 2013; Hickey et al. 2019). Among all food crops, cereals might have been the first to be artificially selected and intentionally planted for food (Meyer et al. 2012; Asano et al. 2011). The domestication process is still conducted though modern breeding techniques, which have completely changed the methods of selection and adaptation of crop traits (Meyer et al. 2012). The major monocots used as daily staples are rice, maize and wheat. In 2018–2019, the production of corn, wheat and rice accounted for approximately 70% of the total world crop production (FAO 2019a; USDA 2019). In the first half of the twentieth century, the world population increased rapidly and disproportionately to the increase in food production, leading to dire predictions for the second half of the century (Khush 2001). We now know that this large-scale famine did not happen due to the first green revolution (GR), which doubled cereal grain production within just 10 years of its beginning in the 1950s. Wheat and rice played major roles in the first GR, indicating the pivotal role of monocot crops in human life.

### Food Requirements in 2050 Vs Present Production: a Major Challenge

The first GR accelerated world food production, which first reached 1 billion tons in 1950, but needed only 10 years to double that number by the use of high-yield varieties, chemical fertilizers and pesticides and the adoption of new cultivation methods involving irrigation systems (Khush 2001). Seventy years after the start of the first GR, the world is again facing the same challenge of feeding a much larger population. Unfortunately, the miracles of the first GR are now reaching their limits. The increases in the yield and production of food crops are slowing and will not meet the requirements of 9.7 billion people in 2050 in the present scenario (UN 2019); for more details, please review the Food and Agriculture Organization (FAO) report (2009) and its revised version published in 2012. A recent report from FAO detailed that the decline in world hunger had reversed in 2015 and that the number of hungry people is slowly increasing at present. As of 2018, over 820 million people are still living under the hunger line (FAO 2019b). It is worth noting that this conclusion was drawn considering the present situation of food production and agriculture, even with worldwide support for conventional and modern molecular-assisted breeding and smart agriculture practices (Ray et al. 2013). Obviously, world agriculture is now challenging, with many novel negative factors, such as more vigorous climate change, soil nutrition deficiency, and global sea level rise, but the other hurdles remain the same as before the first GR. This reality indicates that to cope with the challenges and to fulfill the food production demand, the world must accept and apply new technologies, especially new plant breeding techniques (NPBTs) (Lusser et al. 2011), for crop improvement and agricultural practices (Zaidi et al. 2019). Moreover, the world demands a second GR that can sustain and secure food production for mankind.

### Introduction of NPBTs and Genome-Editing-Based Precision Breeding

**Fig. 1:**
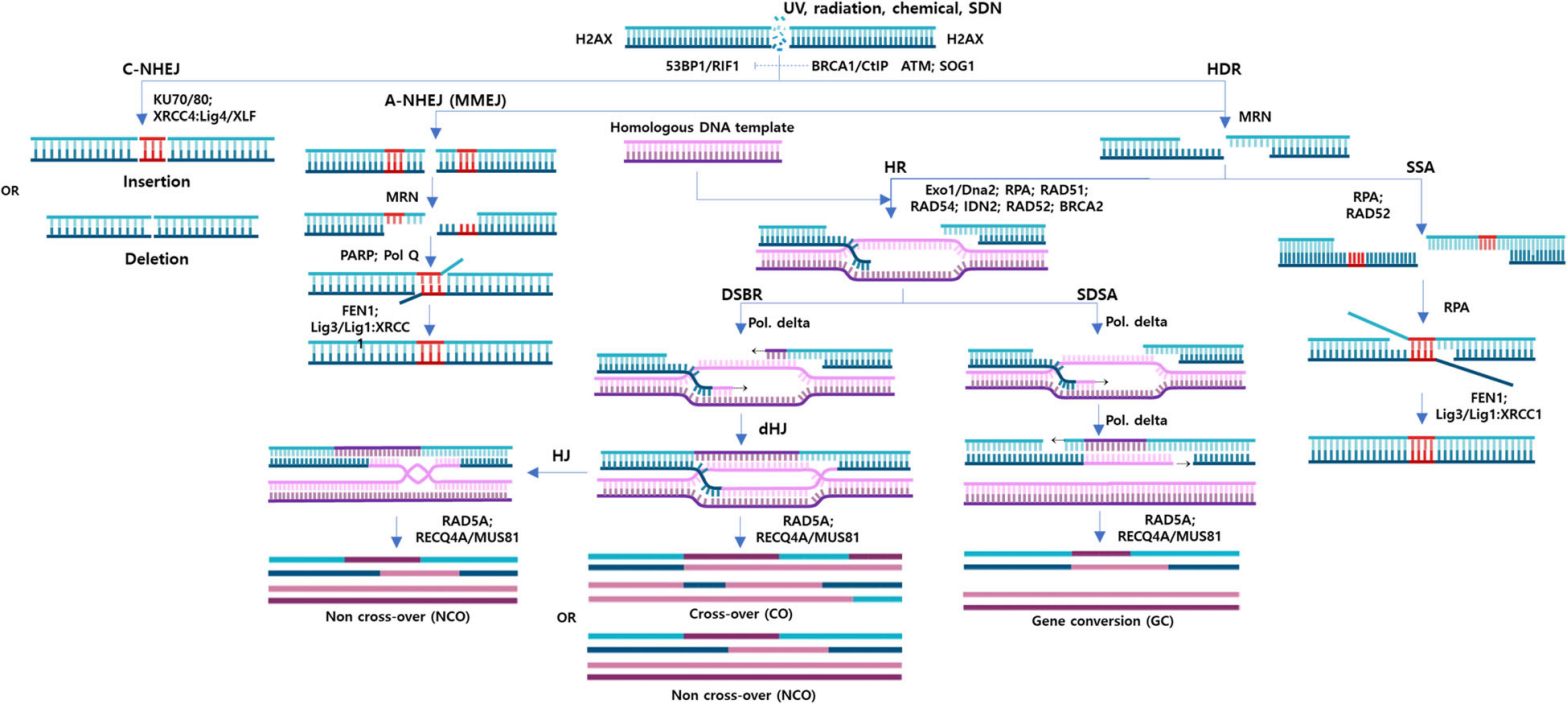
DSB repair pathways.1017In the C-NHEJ pathway, DSB formation induces binding to broken ends by KU70/80 heterodimers that subsequently recruit the DNA damage response kinase (DDK) complex such as DNA-PKcs in mammals. DDK then activates the 53BP1/RIF1 complex, which plays a role in shielding the broken ends from resection by antagonizing BRCA1/CtIP activity. DNA-PK also activates other KU-recruited proteins, such as XLF, XRCC4 and Lig4, for ligating the broken ends. In the HDR pathway, DSB formation induces cell cycle arrest initiated with the activation of ATM resulting from sensing a chromatin structure change. Activated monomeric ATM then phosphorylates the MRN complex and P53/SOG1, which regulates the cell cycle checkpoint and arrest. MRN activation supports end resection for HDR. Limited resection leads to MMEJ, and if a substantial level of resection is formed in the absence of a donor template, SSA is likely to be used for the repair. MMEJ requires PARP and Pol Q for its processes, and SSA requires the role of RAD52. Both MMEJ and SSA require the ssDNA flap endonuclease FEN1 and Lig3/Lig1:XRCC1 for ligating final products. Extensive resection of the broken ends is facilitated by Exonuclease 1 (Exo1) and/or Dna2. In the presence of donor template, the 3′ overhangs of resected ends could be protected by RPA binding and then recruiting RAD51 to the ssDNA with support and control by BRCA2. RAD51 binds to the resected ssDNA overhang, forming nucleoprotein filaments or presynaptic filaments. With the support of RAD54, the filament structure invades the donor template sequence and searches for and anneals to the complementary sequence; then, displacement loop (D-loop) formation occurs. Subsequently, the free 3′ OH end of the invaded ssDNA primes donor template-dependent DNA synthesis. This process determines the outcomes of HDR with several sub-pathways (DSBR with dHJ and SDSA) with the supportive activity of RAD5A, RECQ4A and MUS81. The DNA fragments and protein structures are not pictured to scale. The potential proteins involved in the processes of each pathway or sub-pathway are denoted adjacent to their approaching lines. XRCC: X-ray repair cross-complementing protein; XLF: XRCC4-like factor; Lig4: DNA ligase 4; PARP: poly-ADP-ribose polymerase; Pol Q: DNA polymerase theta

NPBTs, especially the recently emerged genome-editing technologies, offer various solutions to improve crop traits, such as (a) crops that can well adapt to environmental changes resulting in sustainable yield, (b) crops that efficiently use limited resources to produce more food and (c) crops with improved nutritional value. In general, genome editing is a two-stage process that includes (1) DNA damage generation such as single-stranded/double-stranded breaks (SSBs/DSBs) or nucleotide deaminations (for base editors) and (2) host cell repair of damaged sites. The repair process can be error-prone during the canonical nonhomologous end joining (C-NHEJ), alternative NHEJ (A-NHEJ), or single-stranded annealing (SSA) pathways that usually ligate DSB ends without the need for additional DNA templates (Fig. 1). The base excision repair (BER) or nucleotide excision repair (NER) pathways that cells use to fix damaged nucleotides such as deaminated ones may also be error-prone. Other repair pathways such as homologous recombination (HR) or oligonucleotide-directed mutagenesis (ODM) require the presence of homologous DNA donors to replicate genetic information and they have been shown to generate error-free products (Figs. 1 and 3b). Recently, Liu’s team published an exciting novel precision editing approach called ‘prime editing’ which used a reverse transcriptase (RT)-nCas9 (H840A) fusion to precisely add DNA modifications to specific sites (Fig. 3c) (Anzalone et al. 2019). Repair pathways in animals as well as plants have been extensively reviewed elsewhere (Belhaj et al. 2013; Hsu et al. 2014; Doudna and Charpentier 2014; Bortesi and Fischer 2015; Rees and Liu 2018). The NHEJ and BER/NER approaches are highly efficient in generating unpredictable error-prone products, while ODM, HGT and base editor (BE) have been considered as techniques for precision editing of genes in plants. However, in plants, the main obstacle to HGT applications is their extremely low efficacy (Paszkowski et al. 1988; Puchta et al. 1996). Many attempts have been made to improve plant HGT for practical applications. Important enhancements have been shown with CRISPR/Cas complexes with or without homologous donor template delivery and amplification by ssDNA replicons (Baltes et al. 2014; Cermak et al. 2015; Gil-Humanes et al. 2017; Wang et al. 2017). In this review, we summarize recent data regarding genome editing approaches in monocot plants with special focus on HGT and provide perspectives for monocot crop improvement and commercialization.

## Review

### Genome Editing Technologies

#### Targeted Mutagenesis Using Molecular Scissors to Form DSBs

**Fig. 2:**
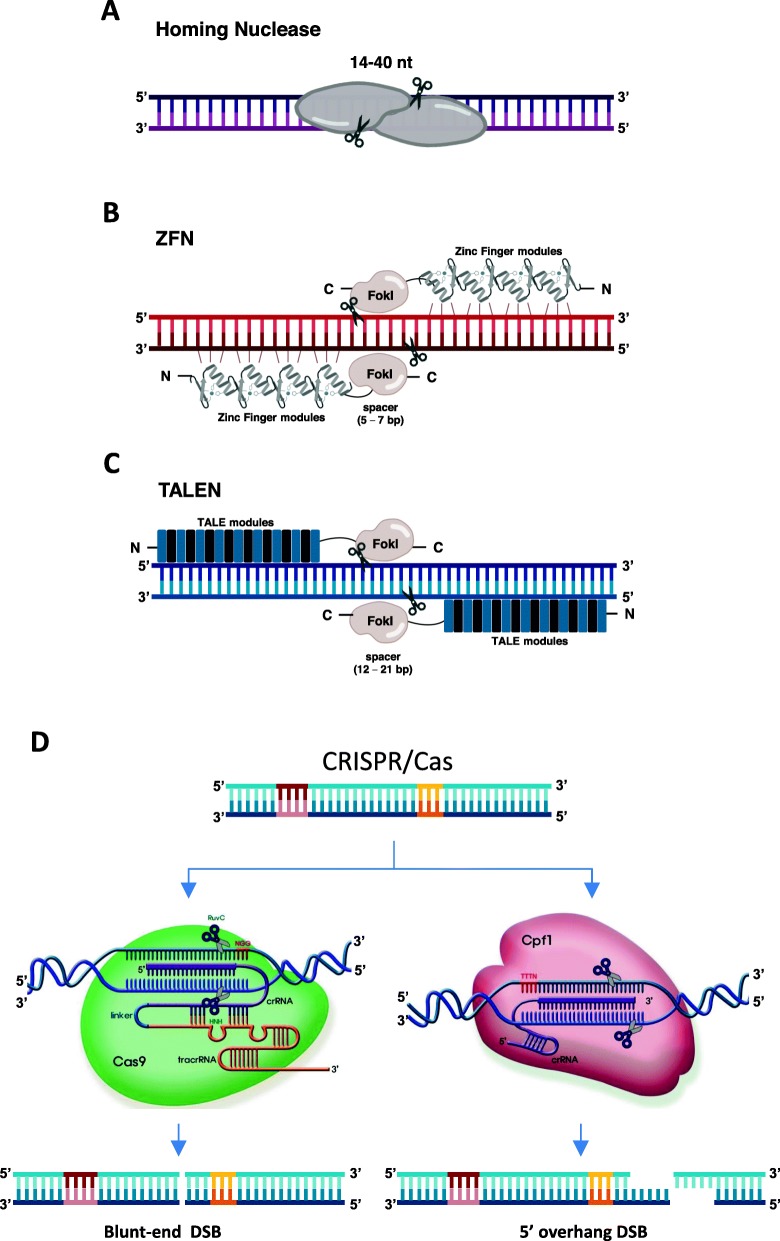
Four generations of molecular scissors. The first, second and third generations of molecular scissors, Homing nuclease (**a**); ZFN (**b**); and TALEN (**c**), are characterized as nucleases relying on DNA binding domains to recognize DNA target sites. Homing nucleases recognize long DNA sequences of 14–40 bp with their DNA binding domains. A ZFN or TALEN is designed by connecting 3–6 zinc finger motifs or 17–20 TALE modules, respectively, for DNA binding and an endonuclease domain of FokI restriction enzyme for cutting. FokI works only in homodimer form, so usually one has to design pairs of ZFNs or TALENs to target a DNA site. FokI activity usually produces DSB with 4 nt overhangs. The fourth generation, CRISPR/Cas (**d**), is also the most powerful one; it uses guide RNA components to form active complexes, thereby interrogating and searching for target DNA sites based on Watson-Crick base pairing between the guide RNA and targeted strand. The DNA fragments and protein structures are not pictured to scale

Since the discovery of restriction enzymes, the field of biotechnology has entered a new era of molecular engineering facilitated by recombinant DNA technology. Several generations of molecular scissors have been discovered, characterized and developed for DSB-based targeted genome mutagenesis. The technology has been improved from the long recognition sequence homing nucleases to protein-dependent DNA binding nucleases, such as zinc-finger nucleases (ZFNs) and transcription activator-like effector nucleases (TALENs), and ultimately to the 3rd generation RNA-guided molecular scissors CRISPR /Cas (Fig. 2). With the invention of target-specific synthetic molecular scissors, the specific modification of a gene of interest in a living organism has become possible. Consequently, there are several key factors involved in targeted mutagenesis induced by molecular scissors, including: 1) the ability to specifically recognize and bind to the targeted DNA sequence, 2) effective DSB formation, and 3) error-prone DSB repair.

The host’s repair of the DSB errors leads to error-free or error-prone outcomes depending on many factors, including the cell cycle state and the availability of homologous DNA templates at the damaged sites. In plant somatic cells, DSB repair by either of the two major pathways, homology-directed repair (HDR) or nonhomologous end joining (NHEJ), usually leads to either error-free or error-prone products. The majority of the error-prone products appear as insertion or deletion (indel) DNA mutations resulting from C-NHEJ or A-NHEJ (Fig. 1). A possibly lower portion of error-prone products may result from SSA repair in the absence of a homologous donor template and from Holliday junction resolution in the last steps of the double-stranded break repair (DSBR) subpathway if the DSB flanking sequences of the sister chromatids are not perfectly matched (Fig. 1). In this section, we briefly summarize the abovementioned molecular scissors. Extensive reviews of the same material can be found elsewhere (Carroll, 2011; Gaj et al., 2013).

##### Generation 0: Homing Nucleases

Homing nucleases are endonucleases (Mw < 40 kDa) that recognize long DNA sequences (14–40 nt) for their cutting activity (Fig. 2a). Homing nucleases can work alone as monomers or in pairs as homodimers (Chevalier and Stoddard 2001). Members of the LAGLIDADG homing endonucleases family such as I-CreI or I-SceI recognize targeted sequences of 22 bp and 18 bp respectively, thus allowing more specific targeting in the host cells (approximately once every 7 × 10^9^ bp) (Jurica et al. 1998; Niu et al. 2008; Chevalier and Stoddard 2001; Jasin 1996). However, this feature also introduces limitations via the scarcity of targetable sites in the genomes of host cells. To compensate for this, researchers have engineered these nucleases for a wider range of binding and cutting sites or combinations of different homing nucleases to recognize multiple sites (Chevalier et al. 2002). Engineered homing nucleases often cleave correct sites as efficiently as wild-type nucleases (Chevalier and Stoddard 2001; Yang et al. 2009; Gao et al. 2010; D'Halluin et al. 2013). However, the engineering of homing nucleases for wider applications is still inefficient, laborious and time consuming.

##### Generations 1 and 2: Protein-Guided DSB Formation, ZFN and TALENs

ZFNs are derived from the discovery of the zinc finger, a finger-like DNA binding motif found in TFIIIA, a transcription factor from the eggs of *Xenopus laevis* (Miller et al. 1985). Its structure comprises 30 repetitive amino acid sequences and is stabilized by a zinc ion (Miller et al. 1985; Berg 1988). Berg (1988) suggested that the zinc finger protein structure might play a key role in the recognition of DNA sequences. ZFN was first developed in 1996 by fusing a nonspecific DNA cleavage domain of FokI, a type II-S restriction enzyme, to the C-terminal of the zinc finger motifs (Kim et al. 1996). Typically, three consecutive nucleotides can be specifically recognized by one zinc finger motif, and therefore, several connected zinc finger motifs fused to FokI can bind the target DNA of interest (Kim et al. 1996). ZFN is the first artificial restriction enzyme that recognizes desirable sites in the genome. Due to their binding specificity and dimerization-dependent FokI activity requirement, ZFNs were typically designed in pairs to recognize 9–18 bp using connected 3–6 zinc finger motifs on both the sense and antisense strands of the targeted sequences spaced by 5–7 bp between ZFNs (Kim et al. 1996; Bitinaite et al. 1998; Laity et al. 2001; Urnov et al. 2010) (Fig. 2b). Post cleavage, the DSB sites were recovered by DNA repair mechanisms that showed insertions or deletions at similar rates (Kim et al. 2013). However, for wider application of this technology, one should overcome the limitations of low editing efficiency (0–24%), elevated design and optimization cost, and high off-target possibility. Many efforts have been made to overcome these barriers. For example, to enhance the cleavage activity of the FokI cleavage domain, Gou and coworkers performed direct evolution to optimize a ZFN named ‘Sharkey’. Several approaches were tested to reduce the off-target effect, e.g., extending the recognition length by using more zinc finger modules (Pattanayak et al. 2011; Guo et al. 2010).

TALEN is the second-generation form of molecular scissors, discovered during studies of the plant immune system under attack and hijacking by pathogenic bacteria (Dangl and Jones 2001). AvrBs3, an effector protein secreted by the plant pathogen *Xanthomonas campestris*, is injected into host cells, thereby binding to the plant UPA-box gene and functioning as a transcription activator to modulate host cell gene expression for its efficient colonization (Kay et al. 2009). The causal agents secreted by *Xanthomonas* were identified and named transcription activator-like effectors (TALEs). TALEs have 33–35 amino acids that are highly conserved, except for those located at positions 12 and 13. These two hypervariable residues (namely, repeat-variable diresidues (RVDs)) are oriented toward the outside of the protein and play a key role in recognizing a specific nucleotide (Moscou and Bogdanove 2009). Common rules of RVD nucleotide recognition for binding were validated as NG for thymine; HD for cytosine; NN for guanine or adenine; and NI for adenine. The first TALENs were introduced by fusing a DNA binding TAL type III effector with a FokI cleavage domain Fig. 2c (Li et al. 2011). However, unlike ZFN, which recognizes 3 bp per zinc finger module, TALENs allow more precise recognition because each RVD of TALE can recognize only one nucleotide. TALENs were designed in pairs with a 12–21 nt distance between two binding sites for the highest cutting activity (Miller et al. 2011). The combination of the TAL effectors AvrXa7, PthXo1 and FokI was demonstrated to function as molecular scissors for cutting and hence modifying the binding sites of the TALs, subsequently resulting in resistance to rice blight disease (Li et al. 2011). The initial NN RVD repeat recognized either guanine or adenine, raising concerns about its specificity (Moscou and Bogdanove 2009). Ultimately, an NK RVD repeat that recognizes only guanine was discovered, fulfilling the specificity requirement for the TALEN molecular scissors (Miller et al. 2011).

One of the weak points of the TALEN approach is the large size of the binding domain, as every nucleotide requires a repeat of ~ 34 amino acids for binding. Thus, to assure high specificity for one TALEN binding to 20 nt, its DNA binding domain must be 680 amino acids long. In addition, assembly of the highly repeated modules was time consuming and laborious. Thus, a well-designed modular RVD repeat library was in high demand and was eventually developed (Zhang et al. 2011; Cermak et al. 2011; Kim et al. 2013). Another limitation of TALENs for practical applications is that they are sensitive to methylated cytosine, thereby preventing them from binding to the modified nucleotide efficiently. In an attempt to overcome the hurdle, the TALE domain was designed to contain the single asparagine RVD (N*) motif (N* refers to Asn instead of Asn-Gly), a base-recognition domain that could effectively bind to 5 ‘methylated cytosine. The engineered TALENs (N*) showed higher efficacies for genome editing in mammalian cells and rice (Valton et al. 2012; Kaya et al. 2017). TALENs have the advantages of high editing efficiency, low off-target activity and lower design cost than ZFNs and the drawbacks of difficult construction, no activity on methylated cytosines (Kim et al. 2013), and difficult introduction into cells owing to their large size (Kim and Ka 2015).

##### Generation 3: CRISPR/Cas

Clustered regularly interspaced short palindromic repeats (CRISPR)/CRISPR-associated protein (Cas) was shown to be a DNA interference-based defense machinery of prokaryotes such as bacteria and archaea against phage infection (Barrangou et al. 2007; Brouns et al. 2008). CRISPR/Cas systems were classified into two classes according to the number of complexity of their effector modules (Makarova et al. 2011; Makarova et al. 2015). Class 1 systems involve effector complexes formed by multiple subunits, whereas in class 2 systems, single multidomain proteins constitute the effector complexes. Furthermore, each class has been divided into several subtypes (class 1: types I, III and IV; and class 2: types II, V and VI) based on their effector architectures with unique signature proteins (Koonin and Makarova 2019). Almost all of the CRISPR/Cas systems used in genome engineering to date are from class 2 due to the simplicity of their effector modules (Additional file 1: Table S1). The most widely used CRISPR/Cas systems are Cas9 and Cas12a (Cpf1).

In the native CRISPR/Cas9 system, phage DNAs were shown to be cleaved by the Cas9 effector complex, which includes the Cas9 protein as a nuclease and a complexed RNA structure formed by a CRISPR RNA (crRNA) and a trans-activating CRISPR RNA (tracrRNA) as a probe. The two-component RNA secondary structure facilitates Cas9 assembly, searching and binding to dsDNA target sites by Watson-Crick complementarity to 19–21 nt of the 5′ end of the crRNA (protospacer) and subsequently cleaving both the strands of the dsDNA at the 3rd nucleotide proximal to a 5′-NGG-3′ protospacer-adjacent motif (PAM) site (Fig. 2d, CRISPR/Cas9). Originally, crRNA:tracrRNA required maturation from a precursor crRNA:tracrRNA by RNase III processing activity, making it more difficult to apply. In the first application of CRISPR/Cas9 for genome editing, the crRNA and tracrRNA were engineered to make a single guide RNA molecule by connecting the 3′ crRNA repeat and 5′ tracrRNA anti-repeat, thereby facilitating the use of the system (Jinek et al. 2012). The Cas9 protein remains inactive until it binds to a guide crRNA:tracrRNA structure. The guide RNA-bound Cas9 complex undergoes conformational changes and then stochastically searches for potential targets by PAM scanning and binding using the PAM-interacting motif. Then, the Cas-sgRNA complex again changes conformation, and the guide RNA sequence is used to pair with the sequence located upstream of the PAM via the Watson-Crick rule (Sternberg et al. 2014; Jinek et al. 2014; Zhu et al. 2019). The gRNA and its seed sequence (10-nucleotide RNA proximal to the NGG PAM) should be fully complemented for R-loop formation and to trigger Cas9 cleavage activities via its endonuclease domains (HNH and RuvC) (Jinek et al. 2012; Jiang et al. 2013; Hsu et al. 2013). The targeted and nontargeted strands of the dsDNA are cleaved by HNH and RuvC, respectively, generating mostly blunt ends (Fig. 2d) (Anders et al. 2014; Nishimasu et al. 2014). Nickase Cas9 (nCas9) that cuts either the targeted strand or nontargeted strand and dead Cas9 (dCas9) were also created by inactivating either the endonuclease domains or both domains for alternative gene editing and regulation.

Unlike Cas9, the Cpf1 system does not require a tracrRNA to mature the crRNA and to form an effector complex for its cleavage activity. The Cpf1 protein was also shown to process the precursor crRNA (Zetsche et al. 2015). After assembly, the Cpf1 effector complex recognizes a T-rich PAM for the initiation of binding and searching for target sites. Its seed sequence was illustrated to range from 1 to 10 nt proximal to the PAM (Kim et al. 2016). The Cpf1 protein has a Nuc nuclease domain that cleaves the target strand and a RuvC domain that cleaves the nontargeted strand (Schunder et al. 2013; Makarova and Koonin 2015; Stella et al. 2017). The nuclease domains cut the target dsDNAs at the 18th nt on the nontargeted strand and the 23rd nt on the targeted strand distal to the PAM, generating 5′ overhang ends (Fig. 2d) (Zetsche et al. 2015).

#### Precision Editing

##### Base Substitution

**Fig. 3:**
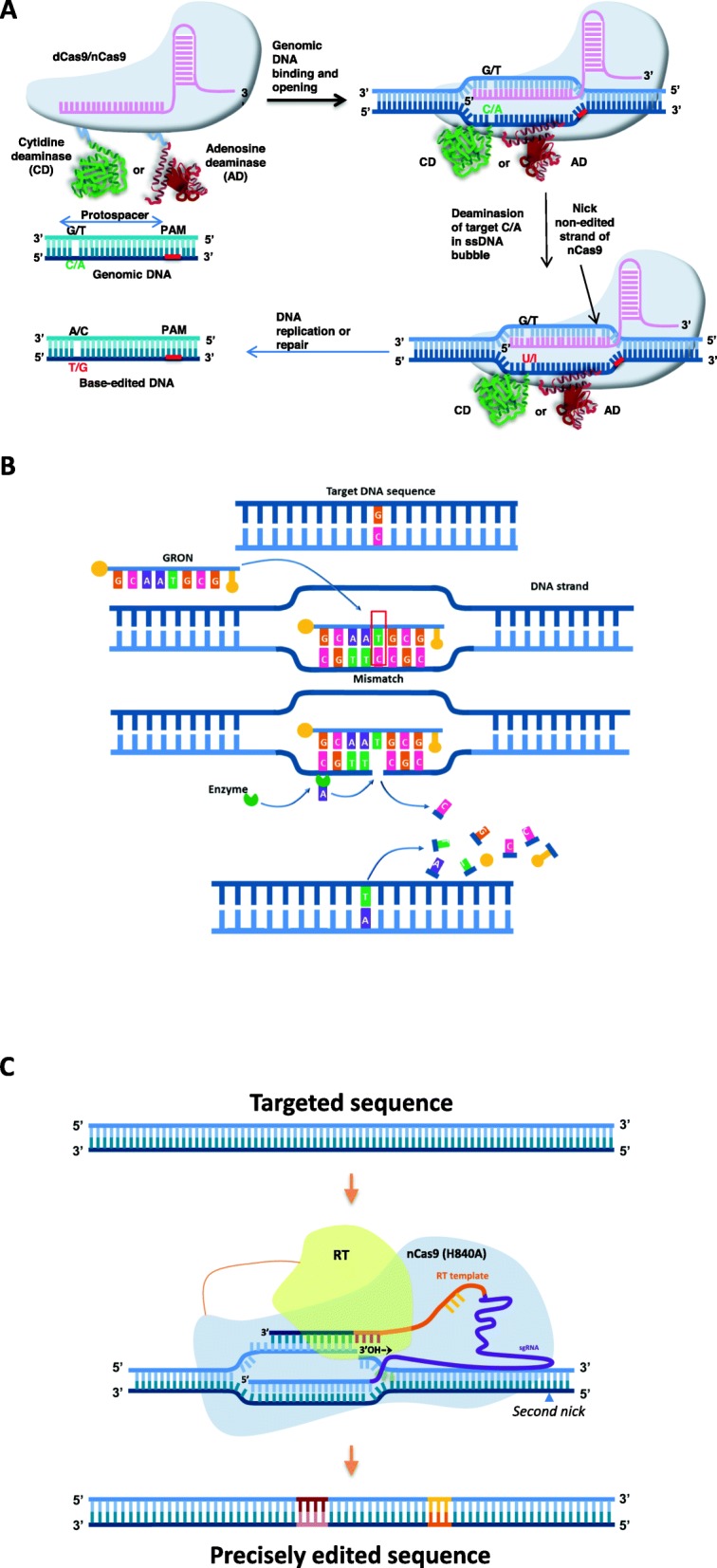
Non-DSB precise gene targeting approaches. **a** Base of approach editing. Cytosine Base Editors (CBEs) and Adenosine Base Editors (ABEs) are the two types of base editors that have been published so far. CBEs: Dead Cas9 (blue) binds to target C (green) via the RNA (pink) guide, which mediates the separation of local DNA strands. A tethered APOBEC1 (green) enzyme by cytosine deamination converts the single-stranded target C to U. The initial G: C is replaced by the A: T base pair at the target location through DNA repair or replication. ABEs: A hypothetical deoxyadenosine deaminase (red) and catalytically impaired nCas9 (Cas9 D10A nickase) bind target DNA in the RNA guide to expose a small bubble of single-stranded DNA that catalyzes the conversion of A to I within this bubble. **b** Oligonucleotide-directed mutagenesis process. A gene repair oligonucleotide (GRON), which contains designed modifications, is delivered and paired with the target DNA sequence. GRON creates a mismatch at the target site and triggers a DNA repair mechanism. DNA repair enzymes detect the mismatch and repair the target DNA sequence using GRON as a template. Once the repair process is completed during cell division and multiplication, the GRON is removed and degraded. The target sequence is modified with designed changes. The representative DNA fragments and protein structures are not pictured to scale. **c** Prime editing. Prime editor is a CRISPR/Cas complex developed by fusion of a reverse transcriptase (RT) to a C-terminal of nickase Cas9 (H840A) and a prime editing gRNA (pegRNA) with a 3 ‘extension that could bind to the 3 ‘nicked strands produced by the nCas9. When bound, the 3′-OH free nicked strand is used as a substratum for the RT to copy genetic information from the 3 ‘extension of pegRNA

It is now well known that the majority of genetic diseases result from point mutations, but the potential DSB-based repair approaches for correcting these mutations are not applicable due to their inaccessibility and the unsuitability of the repair mechanisms (Cox et al. 2015; Hilton and Gersbach 2015). Therefore, a single-base-change technique is highly demanded and has been developed for at least transition fixation (C/G- > T/A or A/T- > G/C): the so-called cytosine base editors (CBEs) or adenosine base editors (ABEs) (Fig. 3a) (Gaudelli et al. 2017; Komor et al. 2016). The basal principle behind the technique is the fusion of dead or nickase Cas9 (d/nCas9) with a cytosine or adenosine deaminase and introduction of the editor complex to the targeted site by the CRISPR guide RNA structure. Deamination of C or A produces U or I, respectively, leading to lesion-by-pass replication and resulting in C/G- > T/A or A/T- > G/C transition, respectively. In addition, the Cas9-based CBEs and ABEs were shown to work in a framed window that was either narrow (13th to 17th nucleotides upstream of the 5′-NGG-3′ PAM) (Komor et al. 2016) or wide (4th to 20th nucleotides upstream of the 5′-NGG-3′ PAM) (Zong et al. 2018) at asymmetric frequency distributions (Additional file 2: Table S2) depending on the types of deaminase used. This fact raises the possibility of controllably and precisely editing every single base of interest by carefully calculating and evaluating the editing frequencies of base editors for a base of a given target. This could also help to avoid the possibility of bystander base changes and unintended off-targets (Gehrke et al. 2018).

##### Oligonucleotide-Directed Mutagenesis (ODM)

Oligonucleotide-directed mutagenesis (ODM) and rapid trait development system (RTDS) are two common names for an oligonucleotide-mediated targeted gene modification technique. This technique uses synthetic oligonucleotides or gene repair oligonucleotides (GRON), which function as a template for endogenous DNA repair to form a heterotriplex with a targeted genome site via homology binding using their sequences, which are identical to the site except at the intentionally modified nucleotide(s), thereby triggering gene conversion and resulting in specific base changes (Fig. 3b). The GRON itself is not inserted into the host genome, and site-directed nucleases or double-strand breaks are not required for this technique. Therefore, ODM was classified as one of the precise gene editing techniques (for review, see Sauer et al. 2016). The changes could be point mutations, multiple base changes, insertions or deletions. The GRON was subsequently degraded during cell divisions, and the modified gene retained its normal pattern of expression and stability within the genome (Sauer et al. 2016).

The first application of synthetic nucleotides was shown in yeast in 1988 (Moerschell et al. 1988) and then in mammalian cells for correction of a faulty human β-globin that causes sickle cell anemia in 1996 (Cole-Strauss 1996; Yoon et al. 1996). In plants, Beetham and coworkers used RNA/DNA chimeric molecules in a work known as the chimeraplasty approach to target tobacco acetoacetate synthase (ALS) (or aceto hydroxyl acid synthesis (AHAS)). Tobacco ALS is a biallelic gene (including alleles ALS1 and ALS2) due to its allotetraploid genome. Therefore, two chimeric ODM oligonucleotides were designed to engineer ALS1 and ALS2 as P196 (CCA) to CAA and to CTA, respectively. Particle bombardment of the oligos and subsequent selection on medium containing 200 ppb of chlorsulfuron revealed one out four ALS alleles with a Pro-196 (CCA) to Thr-196 (ACA) modification. The efficiency was two orders of magnitude higher than that of the control (Beetham et al. 1999). The ODM approach was also conducted in several studies in dicots, such as canola (Gocal 2015) and Arabidopsis (Sauer et al. 2016).

In monocots, ODM was used to target AHAS in maize (Zhu et al. 1999) and rice (Okuzaki and Toriyama 2004). In maize, nucleotide changes were induced at two sites, S621A (AGT to AAT) for imidazolinone and sulfonylurea herbicide resistance and P165A, mimicking the point mutation in tobacco in Beetham’s work (Beetham et al. 1999). The oligonucleotides were transformed into maize cells by bombardment and selected with either 7 μM imazethapyr for S612A or 20 bbp chlorsulfuron for P165A. The mutation frequencies were 1.0 × 10^− 4^ to 1.4 × 10^− 4^, approximately three orders of magnitude higher than that of spontaneous mutation and gene targeting by homologous recombination pathway in plants (Tong Zhu et al. 1999). In rice, three chimeric DNA/RNA oligonucleotides for targeted modification of ALS, P171A, W548 L and S627I, were introduced into rice calli by bombardment. Screening by herbicide selection (chlorsulfuron for P171A and bispyribac-sodium for W548 L and S627I) and Sanger sequencing identified independent transformants for both P171A and W548 L but not S627I at a frequency of 1 × 10^− 4^. The ODM approach was also demonstrated in a wheat system using a transient assay with GFP as a reporter. The authors claimed that using 2,4-D in osmotic media boosted the gene targeting efficiency and that the repair of point mutations had a higher frequency than that of single base deletions in immature wheat embryos (Dong et al. 2006).

ODM products have been considered non-GMOs in a number of countries, although not in the EU, due to the targeted point mutation mechanism and transgene-free outcome (Eriksson 2018). In 2011, the UK Advisory Committee of Releases into the Environment (ACRE) suggested that plants being developed by the ODM system should not be regulated as GMOs. Afterwards, the Federal Office of Consumer Protection and Food Safety of Germany decided that ODM products do not constitute GMOs in 2017. Based on its precise modification and the GMO regulation of this technology, ODM has potential for genome editing. However, its low efficiency is the main barrier for its application in research; thus, improving the editing frequency is essential. Recently, ODM and SDN have been combined to enhance the efficiency with the range of precise editing from 0.09% to 0.23% in an EPSPS target gene. This study also claimed that the transgene targeting efficiency of CRISPR/Cas9 was nearly 3 times higher than that of TALEN (Sauer et al. 2016).

##### HR-Based Gene Targeting

In 1988, gene targeting (GT) or HGT was first defined as modification of the host genome achieved by the integration of foreign DNA via the HR pathway (Paszkowski et al. 1988). This method provides a wide range of targeted genome modifications, such as precise insertion, deletion or replacement of a gene or an allele. In fact, HR is an ideal mechanism that can precisely repair DSBs during the S and G2 phases of the cell cycle, while homologous sequences (sister chromatids or donor templates) are available (Tamura et al. 2002). However, its low frequency in higher plants is still a hurdle for practical applications (Puchta 2005). The first application of HGT in a crop was the targeted knockout of the rice *Waxy* gene using positive/negative selection, which achieved approximately 1% frequency but also left a positive selection marker in the genome (Terada et al. 2002).

Since then, two other important achievements in the plant gene targeting field regarding frequency enhancement have come to light: (1) the key finding of on-target DSB roles (Puchta et al. 1993) and (2) methods to introduce high doses of autonomously homologous donor templates into targeted cells (Baltes et al. 2014). By inducing DSBs at a specific locus using the highly specific restriction enzyme I-*Sce* I, HDR efficiency can be enhanced from 10 to 100 times (Puchta et al. 1996). To further enhance the efficiency of HR for gene targeting, several approaches have been developed. First, site-specific nucleases such as ZFNs, TALENs and CRISPR/Cas systems are applied to induce double-strand breaks at the target sequence (Belhaj et al. 2013; Voytas 2013). The second approach takes advantage of the virus replicon system to increase the delivery ability and the number of donor templates; hence, the HGT efficiency is improved (Baltes et al. 2014). Apart from that, certain studies have demonstrated that overexpression of HR-involved genes or suppression of the NHEJ pathway led to improvement of HGT frequency (Endo et al. 2016; Qi et al. 2013; Shaked et al. 2005).

##### Prime Editing

RNAs were shown to involve in DSB repairs via non-templated or templated mechanisms in human and yeast cells (for more details, see review by Meers et al. 2016). In addition, Butt et al. (2017) successfully engineered the SpCas9 guide RNA scaffold called chimeric single-guide RNA (cgRNA) for acting as sgRNAs and repair templates in rice protoplast. The HGT rate for the replacement of two nucleotides of OsALS locus was shown to be as high as 16.88% of total mutations when plasmids carrying the CRISPR/Cas9 and cgRNA expression cassettes were transfected to the protoplast. Further, targeted insertion of 3xHA tag at the OsHDT701 locus using a cgRNA showed up to 4.69% of total mutations. However, the HGT rates were much lower when only cgRNA-SpCas9 ribonucleoprotein (RNP) complex was transfected while the mutation rates mediated by NHEJ were much higher (Butt et al. 2017). RNA transcripts were further validated as donor templates for HDR-mediated targeting OsALS locus using CRISPR/Cpf1 ribonucleoprotein (RNP) complex. Nonetheless, the HGT frequency obtained with ssRNA donor templates was at 0.07–0.13%, nearly ten folds lower compared to that of ssDNA donors (Li et al. 2019). To expand the use of RNA as templates for plant HGT approaches, further work needs to be done.

Recently, prime editing using guide RNA extensions for priming reverse transcription-mediated precise editing has been shown to be an excellent precision genome editing technique in mammalian cell lines. It would also be an excellent alternative for HGT with a shorter editing sequence coverage. Anzalone and colleagues tested variations in prime editing methods and demonstrated a wide range of specific genetic modifications, including 19 insertions up to 44 bp; 23 deletions up to 80 bp; 119 point mutations, including 83 transversions; and 18 hybrid edits at 12 human and mouse cell lines without explicit DSBs (Anzalone et al. 2019). The prime editor’s best version used a CRISPR / Cas complex developed by fusion of a reverse transcriptase (RT) to a C-terminal of nickase Cas9 (H840A) and a prime editing gRNA (pegRNA) with a 3 ‘extension that could bind to the 3 ‘nicked strands produced by the nCas9. When bound, the nicked strand’s free 3′-OH is used as a substratum for the RT to copy genetic information from pegRNA’s 3 ‘extension (Fig. 3c). If we design pegRNAs to produce modified nucleotides, they would be inserted into the genome during downstream repair processes. A second nick site present downstream of the first nick would support the retention of the de novo nucleotides introduced. (Fig. 3c) (Anzalone et al. 2019). Although prime editor has not yet been used in plant system, we expect this technology to have a bright future in plant genome editing, as plant HGT is much more challenging.

### HGT in Monocots

#### HDR Mechanisms in Plants

One of the principal questions regarding cell response to DSBs is which repair consequences the cells favor: error-free or error-prone DNA products? In meiosis, error-prone crossing over (CO) or break-induced repair (BIR) (or even NHEJ) is preferred for creating genetic diversity by exchanging genetic information between parental chromosomes, a key factor for adaptation to environmental changes. However, we can expect an opposite situation in mitotic cells, which require genetic stability rather than diversity. In that case, should NHEJ be abolished from mitotic cells? The answer is absolutely not, and one of the key reasons may be the limitation of time, because a single DSB persistence may induce programmed cell death after a certain period of time (Nowsheen and Yang 2012). What can the cells do? NHEJ is so abundant and efficient in mending the broken ends. What can we expect from the bulky HDR apparatus?

**Fig 4:**
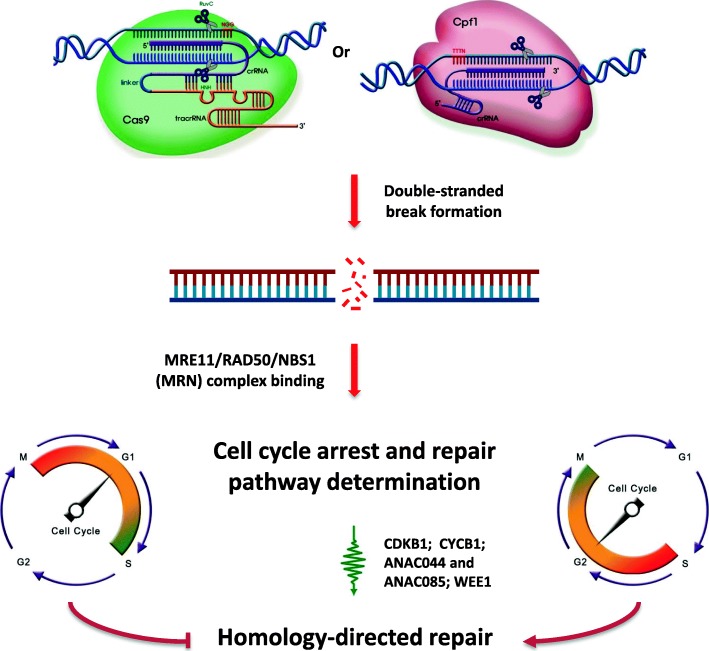
Homology-directed repair pathway determination and its favorable cell contexts. Activation of the MRN complex and P53/SOG1 triggers the activation of cell cycle checkpoint proteins such as CDKB1 (G2/M checkpoint) and CYCB1 (S phase checkpoint) or NAC-type transcription factors ANAC044 and ANAC085 (S/G2 checkpoints) or WEE1 kinase for cell cycle arrest

HDR has been extensively studied in yeasts and mammals for understanding the mechanisms of genetic diseases caused by DNA DSB damage. Most of the components of the plant HDR pathway are homologs of these known proteins (Schuermann et al. 2005), but the regulation of DSB responses in the kingdoms may be different (Yokota et al. 2005). Unlike in animal systems, HDR efficiency in plant somatic cells is extremely low (Szostak et al. 1983; Puchta et al. 1996) and very much dominated by NHEJ. Plant mitotic HDR is absent in the G1 phase and limited to S/G2, while NHEJ is active throughout the cell cycle (Fig. 4). The HDR pathway is determined by the presence of a sister chromatid as a homologous DNA donor, which is normally produced by replication in the S phase and remains present until the M phase. Even in these favorable cell cycle phases, the HDR pathway has to compete with the predominant NHEJ, and hence, it can be chosen in only certain conditions (Heyer et al. 2010; Voytas 2013; Jasin and Rothstein 2013). Therefore, a comprehensive knowledge of the conditions that favor HDR in plant somatic cells would offer key strategies in plant gene targeting for crop improvement.

##### Sensing DSBs and Cell Cycle Arrest

In mammals, DSB formation induces cell cycle arrest, which is necessary to help the cell repair the damage in a reasonable time (Kastan and Bartek 2004). The process is initiated with the conformational changes of the ATM (ataxia telangiectasia mutated) homodimer resulting from sensing a chromatin structure change following DSB formation. Activation of human ATM by autophosphorylation of its serine 1981 disassociates its activated monomers (Bakkenist and Kastan 2003). Monomeric ATM then phosphorylates all the members of the MRE11 (meiotic recombination 11)/RAD50 (Radiation sensitive 50)/NBS1 (Nijmegen breakage syndrome 1) (MRN) complex, a DSB sensor holoenzyme, and is additionally phosphorylated by MRE11 (Lee and Paull 2005; Lamarche et al. 2010). Subsequently, ATM plays a central role in activating cell cycle checkpoint kinases and P53 and indirectly induces the suppression of cyclin-dependent kinases that ultimately leads to cell cycle arrest (Kastan and Bartek 2004; Harper and Elledge 2007; Yata and Esashi 2009). In *Arabidopsis*, SOG1 (SUPPRESSOR OF GAMMA RESPONSE 1), activated by ATM, is responsible for the regulation of multiple downstream proteins such as CDKB1 (G2/M checkpoint) and CYCB1 (S phase checkpoint), the NAC-type transcription factors ANAC044 and ANAC085 (S/G2 checkpoints) or WEE1 kinase for cell cycle arrest (Yoshiyama et al. 2013; Weimer et al. 2016; Takahashi et al. 2019; De Schutter et al. 2007).

##### HDR Pathway Determination

Post DSB formation, cell cycle arrest at S/G2 favors the essential condition for the HDR pathway (Fig. 4). In animals, the HDR pathway is determined by recruitment competition between KU70/80-DNA-PK and the MRN complex to the DSB ends and subsequent resection regulation by BRCA1/CtIP and 53BP1/RIF1, which favors HDR and NHEJ, respectively. However, only the Ku complex but not DNA-PK is conserved in plants (West et al. 2002; Tamura et al. 2002), suggesting an alternative regulation of activation by kinases in the plant kingdom. KU70 was shown to colocalize and interact with MRE11 in somatic cells and therefore was proposed to be a key player in the determination of the DSB repair pathway (Goedecke et al. 1999). Because the majority of DNA end binding proteins in a cell are KU70/80 (Gottlieb and Jackson 1993), NHEJ becomes dominant, and hence, HDR efficiency, especially in plant mitotic cells, is extremely low. Recently, it has been increasingly accepted that DSB end resection plays a key role in the determination of NHEJ- or HDR-mediated repair. NHEJ repair keeps the broken end resection in a limited range for its amendment, but HDR requires DSB end resection to produce 3′-protruding ends that are long enough for template annealing and replication of homologous genetic information. NHEJ resection length usually ranges from 0 to 14 bp, although very rare cases can be 25 bp and longer (Lieber 2010).

ATM-dependent phosphorylation of RAD50, NBS1 and MRE11 of the MRN complex plays an important role in DSB end resection and determines the ultimate repair pathway in an MRN-dependent manner. MRE11 acts as an endonuclease that nicks DNA upstream of the break and subsequently resects 3′- > 5′ toward the break, and then, the end is further resected by Endonuclease 1 and Dna 2 (Kijas et al. 2015). CtIP, activated by ATM, acts in concert with the MRN complex to enhance resection and HDR. CtIP physically interacts with the MRN complex and, more importantly, BRCA1 (Limbo et al. 2007), a protein that inactivates 53BP1 by dephosphorylation (Isono et al. 2017), thereby supporting DSB end resection for HDR determination. However, in a recent study, 53BP1 was shown to shield DSB ends from extensive resection, which might result in a strong bias toward RAD52-dependent error-prone SSA (Ochs et al. 2016). Broken end resection is also controlled by phosphorylated MRE11, which protects exonuclease 1 from extensive resection by phosphorylating it (Kijas et al. 2015). In *Arabidopsis,* PHF11 (plant homeodomain finger 11) plays roles in binding and suppressing RPA, thereby enhancing Exo1 resection (Gong et al. 2017). Furthermore, the resection coordination activity of MRE11 and CtIP/Ctp1 may inactivate KU70/80 and unload it from the broken ends. Meanwhile, a predefined resection length may deactivate the MRN complex and disassociate it from the ends (Langerak et al. 2011).

##### DSB Amendment by HDR

Once the HDR pathway is determined, in the presence of homologous DNA templates, HDR can occur through gene conversion or synthesis-dependent strand annealing (SDSA), single-stranded annealing (SSA) or crossover (CO, DSB repair (DSBR))/noncrossover (NCO) via double Holliday junction (dHj) formation (Holliday 1977). Only the former HDR subpathway can produce precise sequence products (Fig. 1). In plant somatic cells, SDSA was proven to be the major HDR mechanism to precisely repair damaged DNA (Szostak et al. 1983; Puchta et al. 1996; Voytas 2013). The differentiation of HDR subpathways has been well studied in yeasts and mammals but still remains a matter of investigation in higher plants. In the case of HDR, phosphorylation of H2AX histone protein by ATM or DNA-PKcs is important to open nucleosomes for strand annealing. As one H2AX is present for every 10 nucleosomes, efficient HDR requires relaxing up to thousands of base pairs (Lieber 2010). The resection of broken ends at a controllable length of 3′ ssDNA overhangs would favor RAD51-dependent SDSA repair. RPA binds to the resected ssDNAs to prevent the formation of a secondary loop for RAD51 loading. RAD51 loading is facilitated by BRCA2 through its BRC motif, which plays dual roles as an ssDNA-dsDNA junction binding protein as well as a RAD51 docking site provider (Seeliger et al. 2012; Heyer et al. 2010; Dray et al. 2006). The tight regulation of RAD51 loading and nucleofilament formation has been shown to involve a BRCA2-antagonistic protein called FIDGETIN-LIKE-1 (FIGL1) (Fernandes et al. 2018; Girard et al. 2015; Kumar et al. 2019). Extensive end resection with the involvement of Exonuclease 1 (Exo1) and/or Sgs1-Dna2 would lead to RPA disassociation facilitated by RAD52, which redirects to the error-prone SSA repair pathway (Heyer et al. 2010) (Fig. 1).

In *Arabidopsis*, INVOLVED IN DE NOVO2 (IDN2) was shown to help RAD51 loading by binding to RPA and unloading it from DSB ends (Liu et al. 2017). RAD51 binds to the resected ssDNA overhang, forming nucleoprotein filaments or presynaptic filaments. The filament structure invades the donor template sequence and then searches for and anneals to the complementary sequence; this process is followed by displacement loop (D-loop) formation (Rajanikant et al. 2008). RAD54 binds to and is required for supporting RAD51 strand invasion and annealing and for the disassociation of RAD51 afterward (Klutstein et al. 2008; Osakabe et al. 2006). RAD54 formed DNA repair foci in living *Arabidopsis* cells depending on ATM-SOG1 signaling and coincided with the formation of phosphorylated H2AX (Hirakawa et al. 2017). Subsequently, the free 3′ OH end of the invaded ssDNA primes donor template-dependent DNA synthesis. This process determines the outcomes of HDR with several subpathways (DSBR, dHJ and SDSA) depending on the type of DNA synthesis and resolution of the final products (Fig. 1). In the later stage of homologous template-dependent synthesis in somatic cells, the D-loop may be processed and reannealed by the activity of RAD5A, REC4Q and MUS81 (Mannuss et al. 2010; Hartung et al. 2006). Only SDSA can generate precise repair products and is favored in mitotic cells (Heyer et al. 2010; Puchta 2005).

##### HGT in Monocots

Plant gene targeting or HGT was defined by the homology-directed repair (HDR) of an endogenous gene by exogenously introduced homologous DNAs (Paszkowski et al. 1988). Obviously, the initial experiment obtained a very low frequency of homologous recombination (~ 10 ^− 4^), indicating difficulty but feasibility in engineering plant genomes by site-specific gene targeting. Early in the 1990s, a transgenic approach using a preintroduced yeast mitochondrial I-*Sce*I endonuclease as a DSB inducer was adopted in attempts to investigate the mechanisms of DSB repair in plants, especially the HDR pathway in plant somatic cells (Puchta et al. 1993; Fauser et al. 2012; Szostak et al. 1983). It became clear that the HDR pathway employing homologous DNA templates to precisely repair DSB-damaged DNAs occurred mainly via the SDSA mechanism (Fig. 1) with an extremely low efficiency. Nonetheless, the induced DSBs could improve HGT efficiency up to two orders of magnitude (Szostak et al. 1983; Puchta et al. 1996), a large step in plant gene targeting research. Recently, the emerging CRISPR/Cas systems, which have proven to be powerful molecular scissors for in vivo generation of site-specific DSBs, have revolutionized the plant gene targeting approach and brought hope for practical applications in crop improvement.

However, despite the application of flexible approaches (i.e., particle bombardment, protoplast transfection and *Agrobacterium*-mediated transformation) for the delivery and execution of HGT tools, gene targeting in most major crops is still challenging. As mentioned in the previous sections, most of our knowledge about the principal mechanisms of plant HDR has been taken from yeast and animal research studies, and some of those results are inconsistent with observations in the plant kingdom. Therefore, the plant genome engineering community should continuously focus on research to understand plant-specific factors involved in DSB repair, especially via the HDR pathway, the only approach providing precise gene targeting products. Using this background knowledge, one can propose approaches for improving gene targeting frequency. Two of the most important factors affecting gene targeting efficiency in plant somatic cells are 1) DSB formation at the targeted sites and 2) the number of homologous DNA templates available for the sites of breakage (Puchta et al. 1993; Puchta 2005; Townsend et al. 2009; Endo et al. 2016; Baltes et al. 2014).

Because most of the early studies focused on gene targeting in model dicot plants such as *Arabidopsis*, tobacco and tomato (for reviews, see (Voytas 2013; Puchta 2005), monocot gene targeting represented a large gap in the early reports, indicating major challenges in monocot gene targeting. In this section, we aim to summarize recent knowledge regarding gene targeting in the monocot plants that represent most of the major food crops for human beings. In addition, we discuss challenges and suggest potential solutions for improving gene targeting frequency in monocots.

##### HGT without Targeted DSBs

In vivo plant gene targeting without assisted selection was extremely low (Puchta and Hohn 1991; Paszkowski et al. 1988). The first targeted knockout of an endogenous “*waxy*” allele via HGT was successfully generated in rice at a 0.94% frequency by Terada and coworkers (2002) with an innovative positive (hygromycin phosphotransferase II (HptII)-based)/negative (using diphtheria toxin A (DT-A) subunit) selection method (Table 1). The frequency of the gene-targeted *waxy* and *xyl* (b1,2-xylosyltransferase) knockout alleles was further improved by the transformation frequency (Ozawa et al. 2012). The weak point of this strategy is the obligatory use of an associated marker gene; hence, the product is subject to GMO categorization. Therefore, Cre/loxP was applied to excise the marker from the gene-targeted allele (Terada et al., 2010; Dang et al. 2013). The approach was later successfully applied to functional genomic studies via tagging endogenous genes with visible marker(s) (Yamauchi et al. 2009; Moritoh et al. 2012; Ono et al. 2012; Tamaki et al. 2015). The positive/negative system using the DT-A subunit might have posed risks to dicots, because it has not been successfully applied in those plants. Therefore, an alternative positive/negative selection system was developed as an alternative, based on a caffeic acid O-methyltransferase (codA) D314A single-mutated version as the negative selection marker (Osakabe et al. 2014) or neomycin phosphotransferase II (NptII) (positive)/RNAi-based anti-NptII (negative) selection at much lower frequencies (Nishizawa-Yokoi et al. 2015b), which might be a result of less efficient G418 selection in rice. Nonetheless, the positive/negative selection strategy was shown to be unsuccessful in barley (Horvath et al. 2016), highlighting its extremely low efficiency in the absence of DSB and the high genome complexity of monocot gene targeting. In an herbicide-selection-based gene targeting experiment, Endo and coworkers successfully replaced the WT allele of rice ALS with the W548 L and S627I alleles and obtained homozygous T2 plants hypertolerant against an herbicide named bispyribac (BS). Under BS selection, gene targeting occurred at both loci at ~ 3% (Endo et al. 2007). The frequency of targeting OsALS for BS tolerance was enhanced to 6% by using the abovementioned HptII/DT-A selection system, and the selection marker was subsequently excised with the *piggy*Bac system, which can remove a marker gene without leaving a DNA scar (Nishizawa-Yokoi et al. 2015a).

**Table 1:** Major HGT studies in monocots

No.	Gene/Allele	Monocot	HDR tool	Selection marker	HDR allele-associated marker	Homologous donor length (bp) (5′ arm + 3′ arm)	Gene targeting efficiency	Reference	
Gene targeting without targeted DSBs									
1	*waxy* (Granule-Bound Starch Synthase gene knockout)	rice	T-DNA	HptII (positive)/DT-A (negative)	HptII (positive)	6300 + 6800	0.94% (per total surviving calli)	Terada et al. 2002	
2	*Acetolactate synthase* (*ALS*) (W548 L and S627I)	rice	T-DNA	Bispyrobac-sodium (BS) herbicide (positive)	BS herbicide (positive)	8092	~ 3% (per total surviving calli)	Endo et al. 2007	
3	*Alcohol dehydrogenase2* (*Adh2*)	rice	T-DNA	HptII (positive)/DT-A (negative)	HptII (positive)	6200 + 6000	1.9% (per total surviving calli)	Terada et al. 2007	
4	GUS-HptII tagging	rice	T-DNA	HptII (positive)/DT-A (negative)	HptII (positive)	3100 + 3100	~ 5.3% (per total surviving calli)	Yamauchi et al. 2009	
	α-subunit of anthranilate synthase gene (*OASA2*)	rice	T-DNA	Trp-analog 5MT (positive)	Trp-analog 5MT (positive)	7000 in total	0.34% (per total surviving calli)	Saika et al. 2011	
5	Domains Rearranged Methylase 2 (*OsDRM2*), 70 bp targeted deletion	rice	T-DNA	HptII (positive)/DT-A (negative)	HptII (positive)	3000 + 3100	1.9% (per total surviving calli)	Moritoh et al. 2012	
	*OsDRM2*, 3000 bp targeted deletion	rice	T-DNA	HptII (positive)/DT-A (negative)	HptII (positive)	3000 + 3000	0.4% (per total surviving calli)		
6	*Repressor Of Silencing 1a* (*ROS1a*)	rice	T-DNA	HptII (positive)/DT-A (negative)	HptII (positive)	3000 + 3000	1.1% (per total surviving calli)	Ono et al. 2012	
7	*waxy*	rice	T-DNA	HptII (positive)/DT-A (negative)	HptII (positive)	6800 + 5700	1.5% (per total surviving calli)	Ozawa et al. 2012	
	*β1,2-Xylosyltransferase* (*XylT*)	rice	T-DNA	HptII (positive)/DT-A (negative)	HptII (positive)	5500 + 5600	1.8% (per total surviving calli)		
8	*OsRac1*	rice	T-DNA	HptII (positive)/DT-A (negative), Cre/loxP-based removal of HptII	HptII (positive), Cre/loxP-based removal of HptII	3000 + 3000	~ 5.3% (per total surviving calli)	Dang et al. 2013	
	*Caffeic acid O-methyltransferase* (*CAOMT*)	rice	T-DNA	HptII (positive)/CodA (negative)	HptII (positive)	3700 + 6000	~ 12.1% (per total surviving calli)	Osakabe et al. 2014	
9	*ALS* (W548 L and S627I)	rice	T-DNA	BS herbicide (positive) and HptII (positive)/DT-A (negative), piggyBac-based removal of HptII	BS herbicide (positive) and HptII (positive), piggyBac-based removal of HptII	8092	~ 6% (per total hygromycin-resistant calli)	Nishizawa-Yokoi et al. 2015a	
	*cleistogamy 1* (*Oscly1*)	rice	T-DNA	BS herbicide (positive) and HptII (positive)/DT-A (negative), piggyBac-based removal of HptII	BS herbicide (positive) and HptII (positive), piggyBac-based removal of HptII	6000	~ 0.078%		
10	*waxy*	rice	T-DNA	NptII (positive)/RNAi mediated anti-NptII (negative)	NptII (positive)	5000 + 5100	0.26% (per total surviving calli)	Nishizawa-Yokoi et al. 2015b	
	*Glyoxalase I (OsGlb33)*	rice	T-DNA	NptII (positive)/RNAi mediated anti-NptII (negative)	NptII (positive)	5400 + 6200	0.21% (per total surviving calli)		
Targeted DSB-based gene targeting									Site-specific Nuclease
11	*Inositol-1,3,4,5,6-pentakisphosphate 2-kinase* (*IPK*) and *phosphinothricin acetyltransferase* (*PAT*)	Maize	Silicon carbide whiskers-mediated transformation	Bialaphos herbicide (positive)	Bialaphos herbicide (positive)	815 + 815	?	Shukla et al. 2009	Meganucleases
12	Synthetic DNA	Maize	T-DNA	NptII and GFP (positive)	NptII and GFP (positive)	2992 + 1200	0.085% (True event per immature embryo)	Ayar et al. 2013	ZFNs
	*OsPDS*	rice	Bombardment	Transient	Transient	23 + 37 (single stranded oligos)	6.9% (2/29 clones of PCR product, restriction digestion for enrichment of HDR products	Shan et al. 2013	CRISPR/Cas9
13	Pre-intergrated *GFP*	Barley	Bombardment	GFP/YFP fluorescence	GFP/YFP fluorescence	196 + 334	2–3% (per total surviving calli)	Budhagatapalli et al. 2015	TALENs
14	*Acetolactate synthase* (*ALS*) genes (*ALS1* and *ALS2*)	Maize	Bombardment	Chlorsulfuron and bialaphos (positive selection of ALS HDR events).	bialaphos (positive selection of ALS HDR events).	749 dsDNA donor and 127 single stranded oligo donor	0.2% (dsDNA donor) and 0.35% (ssDNA donors)	Svitashev et al. 2015	CRISPR/Cas9
	Upstream of the *LIGULELESS1* (*LIG1*) gene			Bialaphos (positive)	Bialaphos (positive)	1099 + 1035	0.7% (meganulease) and 2.5–4.1 (CRISPR/Cas9)		Meganuclease and CRISPR/Cas9
15	*OsALS* (W548 L and S627I)	rice	Bombardment	BS herbicide (positive)	BS herbicide (positive)	?	1.4–6.3% (per total hygromycin tolerant calli)	Li et al. 2016	TALENs
16	*OsALS* (W548 L and S627I)	rice	Bombardment and T-DNA	Hygromycine and BS (positive)	BS (positive)	100 + 46 (330 bp interval)	7.0–25% (number of perfect HDR events per total calli)	Sun et al. 2016	CRISPR/Cas9
17	*OsALS* (W548 L and S627I)	rice	T-DNA	Hygromycine and BS (positive), *lig4* background	BS (positive)	329 + 635	0.147–1.000% (number of perfect HDR events per total calli)	Endo et al. 2016	CRISPR/Cas9
18	*Nitrate transporter NRT1.1B*	rice	Bombardment	Hygromycine (positive)	None	100 + 100	6.72%	Li et al. 2018a	CRISPR/Cas9
19	*OsALS* (W548 L and S627I)	rice	Bombardment	Hygromycine and BS (positive)	BS (positive)	97 bp left arm only or 97 + 121	0.66% (only left homologous arm) and 1.22% (both arms)	Li et al. 2018b	CRISPR/Cpf1
20	Wheat ubiquitin gene (*TaUbi*)	wheat	WDV replicon bombardment	GFP (positive)	GFP	748 + 773	3.8% (per total protoplasts) and 5.74% (per total cells, normalized to scutela transformation efficiency)	Gil-Humanes et al. 2017	CRISPR/Cas9
	Mildew Locus O (*TaMLO*)	wheat	WDV replicon bombardment	BFP (positive)	BFP	674 + 647	6.4% (per total cells, normalized to scutela transformation efficiency)		
	*5-enolypyruvylshikimate-3-phosphate synthase* (*TaEPSPS*)	wheat	WDV replicon bombardment	dsRED (positive)	dsRED	210 + 646	4.7% (per total cells, normalized to scutela transformation efficiency)		
	*TaUbi* and *TaMLO*, multiplexed	wheat	WDV replicon bombardment	GFP and BFP (positive)	GFP and BFP	748 + 773 (TaUbi) and 674 + 647 (TaMLO)	1.1% (per total cells, normalized to scutela transformation efficiency)		
	*TaUbi* and *TaEPSPS*, multiplexed	wheat	WDV replicon bombardment	GFP and dsRED (positive)	GFP and dsRED	748 + 773 (TaUbi) and 210 + 646 (TaEPSPS)	0.4% (per total cells, normalized to scutela transformation efficiency)		
21	*OsACT* and *OsGST*	Rice	WDV replicon T-DNA	GFP and kanamycin (positive)	GFP and kanamycin	500 + 500	Cas9 overexpressed background: 19.4% (OsACT) and 7.7% (OsGST); WT background 8.5% (OsACT) and 4.7% (OsGST)	Wang et al. 2017	CRISPR/Cas9

##### Targeted DSB-Based HGT

DSBs induced at the gene targeting sites were shown to dramatically enhance efficiency by several orders of magnitude (Puchta et al. 1993). Since the introduced I-*Sce*I meganuclease-mediated DSBs showed significant enhancement of gene targeting frequency, ectopic recombination was tested in maize and revealed remarkably higher efficiencies than the no-DSB strategies particle bombardment and *Agrobacterium*-mediated transformation (D'Halluin et al. 2008; Ayar et al. 2013). However, because the preintroduced homing nuclease targets a predefined sequence in the genome of the plant, gene targeting for a native gene/allele of interest in plant genomes still fell far short of expectations, and site-specific molecular scissors were in high demand. Bearing that in mind, researchers engineered ZFNs, zinc finger motifs for DNA binding fused to the type IIS endonuclease *Fok*I, for efficiently and specifically generating DSBs in vivo (Kim et al. 1996) and obtained significant enhancement of gene targeting efficiency at native loci in *Drosophila* (~ 1.5%) (Bibikova et al. 2003) and human cells (~ 18%) (Urnov et al. 2005). Subsequently, ZFNs were applied to plant gene targeting and yielded an average of 17% HGT efficiency with a preintegrated GUS:NPTII reporter system in tobacco protoplasts (Wright et al. 2005). A similar strategy also obtained ~ 10% HGT efficiency in restoring a preintegrated defective herbicide-tolerance gene (Cai et al. 2009). For targeting multiple allelic loci, also acting as an herbicide-tolerance selection marker, the efficiency was several-fold lower at ~ 2% in tobacco (Townsend et al. 2009). In monocots, ZFN-based gene targeting was first shown to be efficient in maize via integrated insertion of an herbicide-tolerance gene as a selection marker into a native inositol-1,3,4,5,6-pentakisphosphate 2-kinase (IPK) gene (Shukla et al. 2009). Although ZFNs offered a great advantage over meganucleases in plant gene targeting, their design, validation and specificity optimization processes were extremely time consuming and laborious (Puchta and Hohn 2010).

TALENs, the second generation of sequence-specific nucleases, also used protein-based DNA binding domains for targeting sites of interest. Their highly specific and modular binding repeats offered an easier alternative for plant gene targeting. The first plant gene targeting events via HGT using TALENs were in tobacco calli regenerated from protoplasts at 3.5% efficiency without any selection marker (Zhang et al. 2013). Overall, 3.5% of calli showed HGT events without antibiotic selection; however, it is not clear how many protoplasts were used for transfection. The TALEN approach was first applied in monocots to demonstrate the feasibility of gene targeting and reached 2–3% post bombardment of leaves with TALENs plus donors (Budhagatapalli et al. 2015). In rice, a similar range (1.4–6.3%) of gene targeting frequencies was obtained with the OsALS herbicide-tolerance allele (Li et al. 2016).

With the advent of CRISPR/Cas, which revolutionized molecular scissors for DSB formation, plant gene targeting is in theory applicable to any gene/crop of interest due to the simplicity, flexibility and versatility of the system (Jinek et al. 2012; Zetsche et al. 2015). CRISPR/Cas tools have been adapted for wide use in genome engineering studies in various kingdoms, including Plantae (Jinek et al. 2012; Hsu et al. 2014; Barrangou and Doudna 2016). The first attempt to modify a monocot genome via HGT using CRISPR/Cas9 was shown in 2013 by Shan and coworkers. In a transient experiment, OsPDS was modified by HGT at a 6.9% frequency in rice protoplasts using CRISPR/Cas9 for DSB formation and single-stranded oligos as donor templates (Shan et al. 2013). Gene targeting in maize was shown with an efficiency comparison between *Agrobacterium*-mediated delivery and particle bombardment and between a meganuclease and CRISPR/Cas9 at two loci, ALS and LIG1 (Svitashev et al. 2015). Several herbicide-tolerant lines were obtained from the bombardment approach only, indicating a very low targeting efficiency in maize and the requirement of a high dose of donor template and editing tools for enhancing it. The herbicide-tolerance ALS allele was also used in another CRISPR/Cas9-based gene targeting work using a short dsDNA donor delivered as linearized or plasmid forms by bombardment or *Agrobacterium*. With hygromycin and BS herbicide for double selection, the total frequency of HGT events reached 22.5–25%. In detail, most of the HGT lines (42/52) obtained from bombardment showed a range of diversity, with mixtures of perfect W548 L and imperfect S627I. In *Agrobacterium*-mediated delivery, most HGT lines (30/40) were perfect but heterozygous, and the HGT alleles were co-located at the loci with NHEJ alleles (Sun et al. 2016). The highly chimeric HGT patterns indicate prolonged activity of CRISPR/Cas9; unsynchronized states of the cells used in the experiments and/or predominance of organogenesis during shoot formation post editing. Therefore, to synchronize DSB formation and HDR, Endo et al. (2016) used calli stably expressing SpCas9 and sequential transformation of sgRNAs and repair templates for OsALS gene targeting. The HGT frequency was too low, and it was difficult to obtain the target plants. However, when the DNA ligase 4 (LIG4) gene, a key player in the NHEJ pathway, was knocked out before targeting, up to a 1% frequency of gene targeting among the total herbicide-tolerant calli was observed, indicating competition between the NHEJ and HDR pathways (Endo et al. 2016). In an attempt to modify the nitrate transporter gene NRT1.1B using CRISPR/Cas9-based tools, Li and coworkers obtained 6.72% precise replacement of 4 SNPs in the gene sequence without an additional allele-associated selection marker (Li et al. 2018a). The gene-targeted lines might contain DNA insertions in their genome due to the high frequency of DNA integration of the bombardment system, but this possibility was not examined.

An alternative to the Cas9 system is Cpf1-based molecular scissors. The latter cut dsDNAs using a T-rich PAM for binding initiation and usually form 5′ overhangs at their distal ends relative to the PAM (Zetsche et al. 2015). CRISPR/Cpf1 was also used for gene targeting in monocots and showed precise SDSA-based gene replacement at the OsALS loci at comparable frequencies (0.66–1.22%) (Li et al. 2018b) to those of Cas9 systems (Endo et al. 2016).

##### Replicon-Based HDR

Because of the highly efficient replication of geminivirus genomes and their single-stranded DNA nature, these genomes have been used as perfect DNA template cargo for gene targeting in plants. Geminiviral genomic DNAs have been reconstructed to overexpress foreign proteins in plants at up to 80-fold higher levels than those of conventional T-DNA systems (Needham et al. 1998; Mor et al. 2003; Zhang and Mason 2006) due to their highly autonomous replication inside host nuclei and the ability to reprogram cells (Gutierrez 1999; Hanley-Bowdoin et al. 2013). Furthermore, Rep/RepA has been reported to promote a cell environment that is permissive for HR to stimulate the replication of viral DNA (Baltes et al. 2014). Interestingly, it has been reported that somatic HR is promoted by geminiviral infection (Richter et al. 2014). The above characteristics of geminiviral replicons have been shown to make them perfect delivery tools for introducing large amounts of homologous donor templates to plant nuclei. Likewise, the movement and coat proteins of a bean yellow dwarf virus (BeYDV)–based replicon were removed and replaced with Cas9 or TALEN to improve gene targeting in plants (Baltes et al. 2014; Butler et al. 2016; Cermak et al. 2015; Dahan-Meir et al. 2018).

In monocots, wheat dwarf virus (WDV) was first engineered for CRISPR/Cas9-based genome editing and gene targeting in wheat (Gil-Humanes et al. 2017). More importantly, this work showed the feasibility of multiplexed gene targeting of multiple homeoalleles of the wheat genome at a 1% frequency. A similar approach using WDV was also applied in rice for targeted insertion of GFP-2A-NPTII to the C terminals of ACT1 and GST genes in a Cas9-overexpressing WT background. The WDV replicon-based tools showed significantly higher targeted knock-in efficiencies than conventional T-DNA tools (Wang et al. 2017).

##### Present Challenges

Despite higher success rates in gene targeting in plants, most of the abovementioned cases required marker-associated or selectable loci, while the selection and regeneration of HGT events from edited cells are still challenging (Butler et al. 2016; Gil-Humanes et al. 2017; Hummel et al. 2018). The most effective delivery method for HGT tools was reported to be particle bombardment, with relatively high frequencies of gene targeting (see Table 1) due to the high doses of introduced donor DNAs, but it also resulted in multiple DNA integration and/or regeneration difficulties. In addition, compared to other delivery methods, such as *Agrobacterium*-mediated transformation, particle bombardment requires special equipment and costly consumables that are not widely available in every research laboratory. *Agrobacterium*-mediated transformation is a very common and cost-effective method for plant gene targeting, but it showed too low frequencies with conventional T-DNA cargos (Table 1). There has been one solution for delivery of high copy numbers of donor DNAs, without facilitating multiple DNA integration, using autonomous DNA replicons (Baltes et al. 2014; Cermak et al. 2015), but this technique is still challenging in monocots if not used in combination with bombardment (Wang et al. 2017) or with a stable Cas9-overexpressing background and selectable marker (Gil-Humanes et al. 2017). The frequencies were dramatically reduced if multiple allelic loci and/or polyploid plants were targeted (Table 1). Furthermore, the effective application of replicon cargos in plant gene targeting has been shown to be limited by their size (Baltes et al. 2014; Suarez-Lopez and Gutierrez 1997; Gil-Humanes et al. 2017). Therefore, plant gene targeting, especially in cases of nonselectable alleles, is still a matter of improvement.

### Potential Solutions and Perspectives on Monocot HGT

To improve plant gene targeting frequency, understanding HDR mechanisms and finding optimal conditions for HDR are the most important subjects in the field. The initial data on DSB-based gene targeting led to an important conclusion that in plant somatic cells, the majority of HGT-based products were formed via the SDSA pathway (Fig. 1) (Puchta 1998; Voytas 2013; Vu et al. 2017; D'Halluin et al. 2008). Because it is well known that DSB formation is one of the key factors in gene targeting and that viral replicons are used as efficient delivery systems for HDR donor templates, we will discuss and propose only other factors regarding monocot gene targeting here.

#### The Role of Homologous Donor Templates

The initial experiments for understanding plant homologous recombination were mostly conducted in a transient manner using newly introduced homologous DNAs/plasmids in plant protoplasts/cells. Baur et al. (1990) reported extrachromosomal homologous recombinations between two plasmids in tobacco mesophyll protoplasts. The most favorable donor plasmids were in linearized forms that obtained 15- to 88-fold higher recombination efficiency and were proportional to homologous zone size. The closer the break sites were to homologous zones, the higher the recombination frequencies were (Table 2) (Baur et al. 1990). Puchta and Hohn also confirmed that the homologous zone sizes (456 bp to 1200 bp) have a direct correlation with extrachromosomal recombination frequencies in *Nicotiana plumbaginifolia* protoplasts. The frequency was significantly reduced when the homologous zone size was 456 bp or lower (Puchta and Hohn 1991). Single-stranded DNA templates were shown to be efficient substrates for extrachromosomal recombination because they could directly facilitate the initial annealing step between the donor and targeted DNAs. Double-stranded circular DNAs were the least efficient templates for the recombination mode (Bilang et al. 1992; de Groot et al. 1992).

**Table 2:** Potential approaches for improvement of HGT in monocots shown in this review

No.	Category	Approach	HGT mode	Tested in organism	HGT enhancement properties	References
1	homologous repair template	Homologous size and form	Extrachromosomal	*Nicotiana tabacum*	The longer the better; Linearized forms were much better	Baur et al. 1990
2	homologous repair template	Homologous size and form	Extrachromosomal	*Nicotiana plumbaginifolia*	Homologous size was preferred to be more than 456 bp	Puchta and Hohn 1991
3	homologous repair template	Homologous form	Extrachromosomal	*Nicotiana tabacum*	ssDNA was more preferred	Bilang et al. 1992; de Groot et al. 1992
4	HDR-related genes	EcRecA overexpression	Intrachromosomal	*Nicotiana tabacum*	10 folds-efficiency increase with nuclear localized EcRECA	Reiss et al. 1996
5	HDR-related genes	RuvC overexpression	Somatic crossover; Extrachromosomal; intrachromosomal	*Nicotiana tabacum*	Somatic crossover (12 folds); intrachromosomal recombination (11 folds); and extrachromosomal recombination (56 folds)	Shalev et al. 1999
6	HDR-related genes	yeast RAD54 overexpression	SDSA	*Arabidopsis thaliana*	27-fold enhancement	Shaked et al. 2005
7	NHEJ and HDR-related genes	RAD50 knockout	Intrachromosomal	*Arabidopsis thaliana*	8–10 folds of SSA or intrachromosomal recombination	Gherbi et al. 2001
8	NHEJ related genes	AtMLH1 knockout	Intrachromosomal	*Arabidopsis thaliana*	Knockout mutation reduced 72% HDR frequencies	Dion et al. 2007
9	Chromatin modeling	AtFAS1 or AtFAS2 knockout	Interchromosomal	*Arabidopsis thaliana*	40-fold enhancement of somatic homologous recombination	Endo et al. 2006
10	Cell Cycle Synchronization/S phase	Hydroxyurea treatment	SDSA	*Yarrowia lipolytica, Arxula adeninivorans, Saccharomyces cerevisiae, Kluyveromyces lactis* and *Pichia pastoris*	1.2- to 8-fold enhancement	Tsakraklides et al. 2015
11	Cell Cycle Synchronization/S-G2 phases	Cas9 fused with N-terminal (110a.a) of human Gemini	SDSA	Human cell lines (HEK293T)	72% enhancement of somatic homologous recombination	Gutschner et al. 2016
12	Culture conditions	Polyamines (putrescine, spermidine and spermine)	SDSA	Mouse hair follicle model and Human cell lines (U2OS and HEK293)	Polyamines promoted RAD51 loading onto ssDNAs in in vitro assays	Lee et al. 2019
13	NHEJ related genes	Suppression by chemical inhibitors	SDSA	Human cell lines (HEK293T)	KU70 and DNA ligase IV suppression by Scr7 obtained 4–5-fold increase of HR efficiency	Chu et al. 2015
14	NHEJ related genes	Suppression by chemical inhibitors	SDSA	Human cell lines and mouse	DNA ligase IV suppression by Scr7 enhanced CRISPR/Cas9-based HDR frequency up to 19 folds	Maruyama et al. 2015

#### Positive-Negative Selection

Selection and/or regeneration of gene targeting transformants are critical to the success of the approach. The dual mode of selection strongly enhanced the possibility of obtaining gene targeting events in monocots (see Table 1), even without the involvement of the revolutionary CRISPR/Cas molecular scissors. The positive-negative selection system provides a large advantage in rice HGT and may help us improve crops by HGT (Terada et al. 2002). The hurdles in the removal of the associated positive selection markers have been solved by using a smart transposon-based excision system (Nishizawa-Yokoi et al. 2015a). It is exciting to combine the positive-negative selection system with the high DSB performance of CRISPR/Cas complexes for monocot gene targeting.

#### Overexpression of Genes Involved in the HDR Pathway

A good number of HDR-related protein homologs have been identified among prokaryotes and eukaryotes. Attempts have also been made to study and/or improve HDR in somatic cells by overexpressing the proteins in targeted organisms. We discuss these approaches in this section, thereby highlighting important points for the improvement of plant gene targeting frequency.

The *Escherichia coli* RecA protein (EcRecA) was shown to be involved in HR in this bacterium by facilitating ssDNA searching and annealing to its homologous DNA repair templates and subsequently exchanging and displacing the sequence (Radding 1981; Muniyappa et al. 1984; Chen et al. 2008). Overexpression of EcRecA in tobacco protoplasts enhanced the DNA repair efficiency 3-fold upon treatment with interstrand DNA crosslinking agent (mitomycin C) (Table 2). Intrachromosomal HR frequency was also shown to be 10 times higher in cells expressing the protein (Reiss et al. 1996). However, an SpCas9-EcRecA fusion was shown to enhance indel mutation via supporting the SSA repair mode (Fig. 1) and hence to suppress homology-directed gene conversion at 33% in mammalian cells (Lin et al. 2017). In contrast, Cai and coworkers showed a 1.7-fold increase in HGT frequency after cotransfection of the CRISPR/Cas9 complex and EcRecA into human embryonic kidney (HEK) 293FT cells (Cai et al. 2019). In *E. coli*, EcRecA acted in concert with the RuvC protein to resolve Holliday junctions in the late stage of DNA recombination (Iwasaki et al. 1991). By introducing RuvC into the nuclei of tobacco plants, Shalev and coworkers obtained strong enhancement of somatic crossover (12-fold), intrachromosomal recombination (11-fold), and extrachromosomal recombination (56-fold) (Shalev et al. 1999). This improvement may also be useful and applicable for DSB formation-based plant gene targeting approaches.

Activities of helicases have been shown in the initiation of homologous recombination. A transgenic approach using *E. coli* RecQ (EcRecQ) revealed positive effects on extrachromosomal recombination of a two-vector system cointroduced into rice leaves. The EcRecQ transient expression driven by a monocot-specific promoter induced a 4-fold increase in extrachromosomal gene targeting. The stimulation was much higher, at 20–40-fold in cases of stable EcRecQ expression (Li et al. 2004). This report confirmed the importance of helicase activities in HDR and suggested another potential approach for the enhancement of monocot HGT frequency.

As discussed earlier, RAD54 plays roles in concert with the activities of RAD51 during the HDR-mediated DSB amendment stage. Overexpression of yeast RAD54 in *Arabidopsis* was reported to increase gene targeting frequencies up to 27-fold, indicating the importance of strand invasion and/or chromatin remodeling in the HDR pathway (Shaked et al. 2005). The developmental stages of explants used for monocot gene targeting may differentially support the HDR pathway. The largest amount of recombination occurred in embryogenic cells, and this result was explained by the higher expression levels of OsRAD51 mRNA in the cells (Yang et al. 2010). Enhancement of the resection of the broken ends by overexpressing OsRecQl4 (BLM counterpart) and/or OsExo1 (Exo1 homolog) might positively support gene targeting in rice (Kwon et al. 2012).

#### Knockout of Genes Relating to the HDR Pathway

As discussed above, RAD50 plays a central role in the MRN/MRX complex for the resection of the broken ends of dsDNAs. Knockout mutations of RAD50 led to developmental lethality in mice (Roset et al. 2014) and suppression of gene targeting in moss (Kamisugi et al. 2012). Surprisingly, a homozygous rad50 KO *A. thaliana* showed hyperrecombination in somatic cells, as it supported 8- to 10-fold higher gene conversion frequencies of an inverted repeat substrate (Table 2) (Gherbi et al. 2001). This led to an important conclusion that MRN/MRX activities are required by NHEJ more than by HR. The data suggest a strategy that transiently suppresses plant RAD50 during a gene targeting experiment to achieve high frequencies.

Sequence divergence between homologous DNA templates and targeted loci has been shown to affect plant HGT frequency. The HGT frequencies were dramatically reduced by 4.1-, 9.6-, 11.7- or 20.3-fold when the levels of sequence divergence were increased by 0.5%, 2%, 4% or 9%, respectively. The sequence divergence might trigger a nucleotide mismatch repair (NMR) mechanism with the involvement of the NMR key protein AtMSH2 and hence disturb the HDR process (Li et al. 2006; Emmanuel et al. 2006). AtMLH1, a homolog of *E. coli* MutL that is involved in NMR, was shown to be required for homologous recombination and homeologous recombination. AtMHL1 mutation led to strong HDR reduction but a less severe reduction in homeologous recombination (Dion et al. 2007). The data indicate the potential for the regulation of MSH2 and/or MLH1 expression for the enhancement of HGT in monocots, especially when homologous DNA templates with obligate mismatches are used.

Chromosome accessibility is a key factor determining DSB formation and the subsequent repair of the broken DNAs. During replication or transcription, the chromatin is loosened, and the nucleosomes are opened for the assessment of related proteins involved in these processes. The *Arabidopsis thaliana* chromatin assembly factor 1 (CAF-1) complex involved in nucleosome assembly is formed by AtFAS1, AtFAS2 and AtMSI1 subunits. Endo and coworkers showed that knockout mutations of either AtFAS1 or AtFAS2 led to enhancement of somatic HR, potentially by 40-fold, thanks to the opening of nucleosomes for accessibility, cell cycle synchronization favoring HDR conditions, and high expression of HDR-related genes in the mutant backgrounds (Endo et al. 2006). The data suggest a potential enhancement of gene targeting via transient AtFAS1/2 knockdown by RNAi while introducing editing tools in somatic cells of monocots.

Another approach was tested in several studies that showed positive effects on HGT by suppressing important genes involved in the NHEJ pathway, such as KU70/80 or Lig4 (Nishizawa-Yokoi et al. 2012; Endo et al. 2016). This approach also showed a reduction in stable integration of T-DNA in the KU70/80 and Lig4 suppression conditions, suggesting a mechanism of T-DNA integration in the genome.

#### Favorable Tissue Culture Conditions for Gene Targeting

Polyamines accumulated in cells with induced DSBs and were subsequently shown to improve HGT by promoting RAD51-mediated DNA strand exchange. During in vitro assays, polyamines facilitated the capture of duplex DNA by the RAD51 presynaptic filament (Lee et al. 2019). Physical support of the substances may be a good approach for enhancing the activity of RAD51, a key protein in the SDSA subpathway for gene targeting in monocot somatic cells. Chemicals that suppress genes involved in the NHEJ pathway were used for testing HGT enhancement effects. Some chemicals inhibited DNA-PK (Robert et al. 2015) or KU70/80 or Lig4 (Table 2) (Chu et al. 2015; Maruyama et al. 2015), thereby enhancing HGT frequency in mammalian cell lines (Yu et al. 2015). It is still not clear whether we can achieve similar gene targeting enhancement in plants. Data obtained from our laboratory showed nearly no effects of SCR7 and/or RS-1 on tomato gene targeting using geminiviral replicons in combination with CRISPR/Cpf1 (unpublished data). Temperature is an important factor enhancing CRISPR/Cas9-based targeted mutagenesis in plants (LeBlanc et al. 2018) and CRISPR/Cpf1-based HDR in zebrafish and *Xenopus* by controlling genome accessibility (Moreno-Mateos et al. 2017). Recently, we re-engineered geminiviral replicon vectors in combination with CRISPR/Cpf1 and showed enhancement of HGT frequency at high temperatures and under lighting conditions (Vu et al. 2019).

#### Cell Cycle Synchronization

One of the reasons that HDR is limited to the S-G2 phases is the availability of sister chromatids to be used as donor templates. As a consequence, the majority of HDR genes might have evolved to be specifically expressed in these phases. The ideas are to artificially favor cellular conditions (S and G2 phases) in which HDR is more efficient and that limit NHEJ blocking of the targeted sites in other phases (M and G1), especially in the case of Cas9s, because they cut in the core sequences proximal to their PAMs. To that end, cell cycle synchronization at the S/G2 phase using chemical (hydroxyurea) or molecular approaches could be applied (Tsakraklides et al. 2015; Gutschner et al. 2016). Cas9 fused with the N-terminal (110a.a) end of human Gemini, a replication licensing factor that is a direct target of an M/G1-restricted E3 ubiquitin ligase for proteolysis, synchronized Cas9 expression in the S/G2 phase, thereby enhancing HGT up to 87% compared to only Cas9 (Table 2) (Gutschner et al. 2016).

#### *In planta* Gene Targeting

Gene targeting in maize may be performed during fertilization because it provides a permissive environment for sequence exchange by HGT (Djukanovic et al. 2006). In 2012, Fauser and coworkers demonstrated the feasibility of using the pre-integrated target, donor template and homing nuclease (I-SceI) in the planta gene targeting in Arabidopsis to correct the truncated GUS marker in the target with the remaining part located in the donor template. Crossing of the lines carrying homozygous target and donor alleles with a line expressing I-SceI obtained somatic GT events in the F1 generation that could be inherited in the F2 progeny at 6.8 × 10^− 3^ frequency (Fauser et al. 2012). Targeted mutagenesis using CRISPR/Cas9 has been shown to be a highly valuable *in planta* approach for crop improvement (Kelliher et al. 2019). These approaches could also be applied for CRISPR/Cas-based monocot gene targeting, and it would avoid the laborious, time consuming and complex tissue culture process. In planta gene targeting could reduce the mutation rate compared to the tissue culture system, which is accompanied by many mutations. However, the targeting tools should be redesigned to match the conditions (pollen-specific and/or ovule-specific) so that they work within the short time period of pollination and fertilization.

### HDR-Based Monocot Events and Regulatory Aspects

Genome-edited crops, including those created by CRISPR/Cas-based targeted mutagenesis and HGT approaches with or without the uses of DNA cargos, are referred to as products of “new breeding techniques (NBTs)” (Laaninen 2016; Lusser et al. 2011) or “new genetic modification techniques (nGMs)” (Eckerstorfer et al. 2019). In most of these genome-editing events, foreign genetic editing tools could be excluded from the organisms after finishing their roles, except that exotic DNA sequence(s) need to be introduced to specific site(s) in their genome(s). Likewise, most of the genome-edited transformants could not be distinguished among other mutated crops generated by conventional mutagens or natural mutations (Friedrichs et al. 2019; Grohmann et al. 2019), and hence, they should not be regulated. The regulatory legislation seems to be more complicated for HGT events because they have been regulated either as non-GMOs or GMOs by the US, EU, Japan, Australia, and others (for an extensive review, see Eckerstorfer et al. 2019). In this section, we summarize and discuss the regulatory aspects of HGT crops, including monocots, as the critical hurdle for the commercialization of HGT crops. We would also attempt to propose a regulatory principle that could be useful for countries during the legislation process.

#### Current Status in Regulatory Policies for Genome-Edited Crops

The US is the leading country in the commercialization of GM crops to date, with 75 Mha of planted biotech crops in 2018 (ISAAA 2019). In the same year, the US was also the leading country to release policies for the regulation of genome-edited crops. The USDA announced that “Under its biotechnology regulations, USDA does not currently regulate or have any plans to regulate plants that could otherwise have been developed through traditional breeding techniques as long as they are developed without the use of a plant pest as the donor or vector and they are not themselves plant pests” (USDA_Press 2018). This means that genome modifications such as deletions, base substitutions and plant DNA modifications, being similar to those potentially generated by conventional cross-breeding, are all deregulated by USDA policies (NatPlants/Editorial 2018). In Japan, the Ministry of Environment released its final policy on environmental safety on Feb. 8, 2019. According to the decision, creating food items using genome editing is not considered to produce GMOs, under the conditions that any DNA from the nucleases required to edit the target organism are not left within the genome and the resulting gene edits could have also occurred naturally. The Japanese Ministry of Health, Labor and Welfare announced a nearly identical assessment with regard to food safety on March 27, 2019 (USDA/JA9050 2019). Brazil, Argentina, Canada, Chile and Colombia have decided to regulate genome-edited crops at similar levels to the US (Ledford 2019). The Australian government adopted a middle level of regulation because SDN-1 products would not be regulated (Mallapaty 2019). By contrast, on July 25, 2018, the European Court of Justice decided that genome-edited crops would be subject to the same rules as transgenic plants or animals (ECJ 2018). Other governments, including those of the Republic of Korea, China, Russia and India, are still making their determinations of how to regulate this technology.

#### SDN Declaration

In fact, according to the released ruling policies of the governments except the EU, not all genome-edited transformants are considered non-GM. In principle, the genome-edited crops were initially divided under the classification of the so-called site-directed nucleases (SDN) by the European Food Safety Authority (EFSA) in 2012: “In SDN-1 applications, only the SDNs are introduced into plant cells (stably or transiently), generating site-specific mutations by nonhomologous end-joining (NHEJ). In SDN-2 applications, homologous repair DNA (donor DNA) is introduced together with the SDN complex to create specific nucleotide sequence changes by homologous recombination (HR) or homology-directed repair (HDR). The SDN-2 technique can introduce substantial changes to the nucleotide sequences of the target gene but more precise changes according to the bioengineer’s plan. SDN-2 techniques can provide unlimited SNP alleles that can boost innovative crop breeding. In the SDN-3 technique, a large stretch of donor DNA (up to several kilobases) is introduced together with the SDN complex to target DNA insertion into a predefined genomic locus. The predefined locus may or may not have extensive similarity to the DNA to be inserted. The insertion can take place either by HR or by NHEJ. In the case of insertion by means of NHEJ, the technique is denominated the SDN-3–NHEJ technique” (EFSA 2012). This classification is now generally accepted as the basal information for genome-edited crop regulation. On August 20, 2018, Japan’s Ministry of Environment (MOE) released a draft of its regulatory policies, adding some detailed requirements for SDNs to be excluded from the Cartagena Protocol regulation (USDA/JA8064 2018). The levels of regulation are decided based on the presence/absence of foreign genetic carriers, the levels of modification and the natural existence of the modification in genome-edited organisms. From another point of view, they are assessed on a case-by-case basis (see Table 3). From the released regulations, it is now clear that HGT will be regulated as either non-GMO (some cases of SDN-2) or GMO (some cases of SDN-2 and SDN-3).

**Table 3:** Gene- edited crop regulation status on the basis of SDN

Category	EFSA-2012 Definition	The major contents of Regulation			
		USA	Japan	Australia	EU
SDN-1	After the intended site-specific cleavage of the DNA in the genome, random mutation (base substitution, insertion, or deletion) occurring for one or a few bases as a natural repair mechanism	Excluded from regulation if the resulting plants are free of DNA from “plant pests” such as viruses or bacteria	When there are no transgenic genes and/or fragments of transgenic genes in the final product, however, the genome edited foods will not be considered to be foods derived from recombinant DNA technology, as long as, the DNA double-strand break induced by engineered restriction enzyme and following repair (i.e., mutation) is: a) base-pair deletion; b) substitution; c) naturally occurring gene deletion; and/or, d) concomitant insertion (mutation) of one to several base pairs.	Excluded from regulation	Regulated as GMO(s)
SDN-2	Systematically induces mutation for one or a few bases by artificially synthesizing a short DNA fragment (template) that is homologous to the target base sequence and introducing it along with an artificial restriction enzyme at the time of cleaving.			Explicitly regulated	Regulated as GMO(s)
SDN-3	Forms a special DNA fragment at a specific domain on the genome by introducing a long DNA fragment containing a gene of several thousand base pairs not originating from compatible same or related varieties (transgene) in a form sandwiched by sequences homologous to the target sequence.			Explicitly regulated	Regulated as GMO(s)
Effective date		28 March 2018	27 March 2019	8 October 2019	25 July 2018

The classification and regulatory considerations have created a major challenge for plant gene targeting approaches to be commercialized, even though their efficacy would be enhanced at a practical level. Gene targeting for modifying SNPs is deregulated by “relaxed” governments such as the US but not Australia. HGT products subject to the SDN-3 category, containing inserted sequence(s) that could not potentially form in nature, will all be regulated as transgenic products (Table 3).

#### A Regulatory Proposal for NBPT Products

Many governments seem to be trying to create sufficient oversight to protect the public interest and at the same time not create new obstacles to technical innovation. Genome-editing-based precision breeding is an innovative technology, but the technologies will evolve continuously. In particular, HDR-based precision breeding technologies are the most cutting-edge technology among genome-editing techniques because they can produce both precise SDN-1 and SDN-2/SDN-3 products. HDR-based precision breeding products are generally classified as SDN-2 or SDN-3, as they use a DNA donor template during the gene editing process and are thus regulated as GMO in Australia and Japan, with potential exceptions. Mechanical classification of HDR-based genome-edited products in the GMO category might pose the most unreasonable obstacle to this plant breeding innovation. In fact, HDR-based precision breeding can fulfill the long-awaited dream of breeders by precisely introducing beneficial gene alleles from crossable relatives without other trait compromises, such as linkage drag (mixing of targeted beneficial traits and unintended undesirable traits by linkage effects). What might be the solution? We must now again remind ourselves of the purpose of the regulation of biotechnology products. Regulations exist to prevent new products from harming human health or the environment. Many various technologies can be used to produce the same or similar, effectively indistinguishable, products to traditional breeding products; therefore, the consistent risk-based regulatory approach is to treat similar products identically. In this view, it is worth referring to the Canadian regulatory policy, which regulates only plants with novel traits (PNTs), irrespective of the technologies used (Ellens et al. 2019). According to Canadian regulation, some SDN-1 products or even chemically mutagenized products can be regulated. However, this regulatory policy provides more open opportunities to use various innovative technologies, including genome editing or GMO. In the end, the fruitfulness of NPBT crops will mainly depend on the level of regulation on NPBT products.

## Conclusions

The development of novel traits for monocot crops is crucial for coping with future challenges in crop production for feeding approximately 10 billion people in 2050 (Hickey et al. 2019; Ray et al. 2013). The recent development of NPBT has paved ways for crop improvement to cope with difficult missions. Targeted mutagenesis approaches (Fig. 1, error-prone approaches, and Fig. 2) of NBPT have been gaining significant success in targeting a wide range of crop plants, including monocots, due to the ease and high frequencies of the revolutionized molecular scissors, especially the CRISPR/Cas complexes (Fig. 2 and Additional file 1: Table S1). Similar outcomes could also be expected with base-editing techniques (Fig. 3b and Additional file 2: Table S2). However, precision editing approaches such as ODM (Fig. 3a) and especially plant HGT approaches are still facing hurdles in practical application due to their low efficiencies and the complexities of editing event regeneration (Fig. 1, HR and SDSA approaches, and Table 1). A significant number of studies have been conducted to unveil the mechanisms of the HDR pathway (Fig. 1) and to overcome the obstacles in HGT frequency (Table 2). The major HGT support strategies to date are (1) appropriate selection of HGT events; (2) enhancement of DSB formation frequency and specificity at the targeted site; (3) high-dose delivery of homologous donors; (4) overexpression/interference of genes involved in HR/C-NHEJ; (5) chemical-based activation/suppression of genes involved in HR/C-NHEJ; and (6) chemical/biological-based cell cycle synchronization (Table 2). Although it is still not clear, we realize the potency of the in planta HGT approach, which helps to avoid the laborious and time-consuming tissue culture process. In addition, it may reduce unintended effects due to the high performance of the CRISPR/Cas system as well as genetic variation in callus-mediated plant regeneration. Appropriate applications of each of these strategies alone or in combination and with the use of CRISPR/Cas complexes may offer a better way to overcome the low efficiency and regeneration concerns. More work still needs to be done for practical customization of monocot crop traits using the HGT technique.

Recently, negative predictions about food production have forced several governments to accept NPBTs as the only way to sustain our future. Regulatory legislation has been more relaxed, with NPBT products produced by SDN-1 and SDN-2 (case-by-case) in the USA, Japan and Australia but not the EU. Countries with pending regulations include China, India, and the Republic of Korea. Based on this background and understanding, we have attempted to propose a few principles for upcoming regulatory policies for these countries.

## Additional Files

**Additional file 1: Table S1.** Targeted mutagenesis-genome editing in monocots using CRISPR/Cas.

**Additional file 2: Table S2.** Major applications of base-editing approaches in plants.

## Data Availability

Not applicable.

## Abbreviations

A-NHEJ: Alternative nonhomologous end joining
BER: Base excision repair
Cas: CRISPR-associated protein
C-NHEJ: Canonical nonhomologous end joining
CO: Crossover
CRISPR: Clustered regularly interspaced short palindromic repeat
dHj: Double Holliday junction
DSBR: Double-stranded break repair
DT-A: Diphtheria toxin A
FAO: Food and Agriculture Organization
GR: Green revolution
GT: Gene targeting
HDR: Homology-directed repair
HGT: Homology-directed gene targeting
HptII: Hygromycin phosphotransferase II
HR: Homologous recombination
MMEJ: Microhomology-mediated end joining
NCO: Noncrossover
NER: Nucleotide excision repair
NPBTs: New plant breeding techniques
NptII: Neomycin phosphotransferase II
SDN: Site-directed nucleases
SDSA: Synthesis-dependent strand annealing
SSA: Single stranded annealing
TALENs: Transcription activator-like effector nucleases
ZFNs: Zinc-finger nucleases
